# Explaining cognitive function in multiple sclerosis through networks of grey and white matter features: a joint independent component analysis

**DOI:** 10.1007/s00415-024-12795-2

**Published:** 2025-01-15

**Authors:** Senne B. Lageman, Amy Jolly, Nitin Sahi, Ferran Prados, Baris Kanber, Arman Eshaghi, Carmen Tur, Cyrus Eierud, Vince D. Calhoun, Menno M. Schoonheim, Declan T. Chard

**Affiliations:** 1 NMR Research Unit, Queen Square MS Centre, Department of Neuroinflammation, Faculty of Brain Sciences, UCL Queen Square Institute of Neurology, University College London, Queen Square, London, WC1N 3BG UK; 2 Alzheimer Center Amsterdam, Neurology, Vrije Universiteit Amsterdam, Amsterdam UMC Location VUmc, Amsterdam, The Netherlands; 3 Department of Medical Physics and Biomedical Engineering, Centre for Medical Image Computing, UCL, London, UK; 4 e-Health Centre, Universitat Oberta de Catalunya, Barcelona, Spain; 5 Multiple Sclerosis Centre of Catalonia (CEMCAT), Vall d’Hebron Barcelona Hospital Campus, Barcelona, Spain; 6 Tri-Institutional Center for Translational Research in Neuroimaging and Data Science (TReNDS), Georgia Institute of Technology, Georgia State University, Emory University, Atlanta, GA USA; 7 MS Center Amsterdam, Anatomy and Neurosciences, Vrije Universiteit Amsterdam, Amsterdam Neuroscience, Amsterdam University Medical Centers, Location VUmc, Amsterdam, The Netherlands; 8 National Institute for Health Research (NIHR), University College London Hospitals (UCLH), Biomedical Research Centre, London, UK

**Keywords:** Joint independent component analysis, Multiple sclerosis, Cognition, Grey matter, White matter

## Abstract

Cognitive impairment (CI) in multiple sclerosis (MS) is only partially explained by whole-brain volume measures, but independent component analysis (ICA) can extract regional patterns of damage in grey matter (GM) or white matter (WM) that have proven more closely associated with CI. Pathology in GM and WM occurs in parallel, and so patterns can span both. This study assessed whether joint-ICA of GM and WM features better explained cognitive function compared to single-tissue ICA. 89 people with MS underwent cognitive testing and magnetic resonance imaging. Structural T1 and diffusion-weighted images were used to measure GM volumes and WM connectomes (based on fractional anisotropy weighted by the number of streamlines). ICA was performed for each tissue type separately and as joint-ICA. For each tissue type and joint-ICA, 20 components were extracted. In stepwise linear regression models, joint-ICA components were significantly associated with all cognitive domains. Joint-ICA showed the highest variance explained for executive function (Adjusted *R*^2^ = 0.35) and visual memory (Adjusted *R*^2^ = 0.30), while WM-ICA explained the highest variance for working memory (Adjusted *R*^2^ = 0.23). No significant differences were found between joint-ICA and single-tissue ICA in information processing speed or verbal memory. This is the first MS study to explore GM and WM features in a joint-ICA approach and shows that joint-ICA outperforms single-tissue analysis in some, but not all cognitive domains. This highlights that cognitive domains are differentially affected by tissue-specific features in MS and that processes spanning GM and WM should be considered when explaining cognition.

**Supplementary Information:**

The online version contains supplementary material available at 10.1007/s00415-024-12795-2.

## Introduction

Multiple sclerosis (MS) is an inflammatory demyelinating disease of the central nervous system (CNS), characterized by white matter (WM) and grey matter (GM) lesions and atrophy, with widespread neuro-axonal loss [1]. MS can affect numerous CNS functions, but typically presents with motor or sensory symptoms attributable to brain WM, spinal cord or optic nerve lesions. However, if assessed, approximately 40–70% of people with MS have cognitive impairment (CI). Information processing speed, working memory, visuospatial ability, and executive function are commonly affected, although the pattern of CI is heterogeneous across individuals, even within the same clinical phenotype [1–3]. CI can severely impact a person’s quality of life and psychosocial function and is a significant factor associated with unemployment. Yet, CI in MS remains only partly explained by current models seeking to link observed brain pathology with outcomes [4–6].

Magnetic resonance imaging (MRI) measures that are derived at a whole-brain level, for example, the quantification of total WM lesion volume and global GM brain atrophy, are only partially associated with CI [7, 8]. Given MS disease effects are not homogeneous across the brain [9, 10], and different cognitive functions are supported by distributed but overlapping neural networks, it is unsurprising that the explanatory ability of whole-brain measures is limited [11–13]. Using network-based approaches can improve the ability of MRI measures to explain outcomes and has shown promise in helping understand CI [14, 15]. For example, independent component analysis (ICA), which identifies covarying patterns within data, has been successfully used to identify networks of GM atrophy in MS. Overlapping regional patterns of cortical atrophy corresponding to the default mode network (DMN) and other known functional networks were associated with cognition [10]. More recently, using a similar data-driven ICA approach, it has been found that some GM components, including a cortical–basal ganglia-like network, are associated more closely with cognitive disability compared to global GM atrophy, deep GM atrophy and lesion volumes [14]. Furthermore, ICA GM patterns, including a salience network-like component, better predict cognitive worsening over time compared to whole-brain MRI measure models. A WM ICA study using tract-based spatial statistics (TBSS) has also found a relationship with cognitive function. This study extracted components of microstructural integrity loss in WM tracts, such as the supratentorial projections and limbic association tracts, which were found to be associated with executive function and visuospatial memory, respectively [16].

Studies combining GM and WM have shown that both tissues provide complementary information. While GM and WM pathologies may occur independently (e.g., the accrual of lesions, which are not closely related in GM and WM [17]), WM and GM are also interrelated, for example, damage in WM tracts is associated with atrophy in connected brain regions, particularly in RRMS(9). This suggests that when seeking to explain clinical outcomes, GM and WM pathology should be considered together, allowing for relationships between them. Yet to date, ICA studies in MS have typically considered GM or WM in isolation. Parallel ICA jointly extracts components from two modalities and then assesses the correlations between them. The power of this approach has recently been demonstrated in an MS study investigating GM atrophy and WM lesion distribution using parallel-ICA [18]. This study found that components of WM lesions correlated with components of GM atrophy, and the WM lesions components could predict progression of motor function decline in people with relapsing–remitting MS (RRMS). However, this study did not assess other clinical outcomes, such as cognition, or include people with primary progressive MS (PPMS) or secondary progressive MS (SPMS), limiting the generalizability of the findings. Furthermore, while parallel-ICA can identify relationships between GM and WM networks of pathology, it identifies links between separately extracted features rather than directly isolating components with shared GM and WM features: joint-ICA is a multivariate approach that is able to address this, as it jointly estimates multimodal components covarying across participants, which are captured in a shared loading matrix despite the data being measured with different techniques. A single linkage parameter is subsequently determined for every component and subject. This way, joint-ICA provides a unit-free approach to characterize latent factors that connect datasets of entirely different scales. This method has previously been applied to GM volume and WM microstructure measures in studies on dementia, bipolar disorder, and obsessive–compulsive disorder, and has shown that joint-ICA components represent distinct shared patterns of GM and WM pathology in the brain [19–21]. These studies also found that such shared patterns were associated with cognitive function.

In the present study, we applied joint-ICA to GM volumetric and WM diffusion-weighted imaging (DWI) data. We chose these two MRI measures as they reflect pathology mediated through neural networks, specifically neuronal loss (GM volume) and axonal degeneration (WM DWI measures). We hypothesized that combining patterns of GM volume and WM integrity using this data-driven multivariate approach would, compared with single-tissue ICA, improve our ability to explain heterogeneity in cognitive function in MS. To test this hypothesis, we used data from a diverse cohort of people with RRMS, SPMS, and PPMS to: 1) establish if patterns of GM and WM changes in MS can be directly linked through joint-ICA, and if so, whether these can be linked with known functional brain networks; 2) investigate whether specific joint-ICA components have differential relationships with cognitive domains and; 3) determine if joint-ICA can increase the explained variance in cognitive function compared to single-tissue GM and WM ICA.

## Methods

### Participants

Eighty-nine people with MS were included in this study. Participants were recruited between 2010 and 2013. Inclusion criteria were; a diagnosis of MS of any type or duration, age between 18 and 65 years, the ability to give written informed consent and have an MRI scan. Exclusion criteria were; any other known neurological disease or medical condition that could affect the brain, pregnancy or breastfeeding. There were no clinical relapses or steroid treatments in the 30 days prior to assessment of all participants. The cohort’s demographics are summarized in Table 1. Participants had a diagnosis of clinically definite MS according to the 2005 McDonald criteria [22]. MS subtypes were classified using the Lublin–Reingold criteria [23]. All participants gave written informed consent. This study had research ethics committee approval (09/H0716/77).

**Table 1:** Demographics, clinical and cognitive characteristics

	All MS	RRMS	SPMS	PPMS
Demographics				
*N*	89	53	22	14
Age (years)	46.3 (± 10.7)	41.9 (± 10.1)	54 (± 7.24)***	50.9 (± 8.99)**
Sex (male:female)	29: 60	16: 37	7: 15	6: 8
Clinical characteristics				
Duration (years)	14.7 (± 9.77)	11.6 (± 7.93)	24.3 (± 9.11)***	11.5 (± 7.42)^###^
EDSS (median[range])	4.5 [1–8.5]	2 [1–7]	6.5 [4.5–8.5]***	6 [1.5–6.5]**^#^
Current DMT use (*n*) (current/previous/never)	44/7/38	36/3/14	8/3/11	0/1/13***^#^
Cognitive characteristics				
IPS	0 (± 1.00)	0.29 (± 1.02)	-0.54 (± 0.84)**	-0.24 (± 0.80)
Vermem	0 (± 0.87)	0.15 (± 0.80)	-0.21 (± 0.80)	-0.23 (± 1.13)
Vismem	0 (± 1.00)	0.22 (± 0.92)	-0.51 (± 1.04)*	-0.03 (± 1.05)
EF	0 (± 0.59)	0.10 (± 0.54)	-0.15 (± 0.63)	-0.14 (± 0.69)
Workmem	0 (± 1.00)	0.16 (± 1.04)	-0.30 (± 0.88)	-0.11 (± 1.01)

Data are presented as mean with standard deviation unless mentioned otherwise. Cognitive characteristics are presented as z-scores (based on the cohort values) averaged across all tests relating to each cognitive domain. Differences between groups were compared for age by ANOVA and Tukey HSD post-hoc, for sex by Chi-square test, for disease duration and EDSS by Kruskal–Wallis tests and Wilcoxon rank-sum tests post-hoc and for cognitive domain scores by ANCOVA with age as covariate and Tukey HSD post-hoc when appropriate. *p < 0.05, **p < 0.01, ***p < 0.001 compared to RRMS. ^#^*p* < 0.05 ^###^*p* < 0.001 compared to SPMS. *DMT* disease-modifying therapy, *EDSS* Expanded Disability Status Scale, *EF* executive function, *IPS* information processing speed, *MS* multiple sclerosis, *N* sample size, *PPMS* primary progressive multiple sclerosis, *RRMS* relapsing–remitting multiple sclerosis, *SPMS* secondary progressive multiple sclerosis, vermem—verbal memory, vismem—visual memory, workmem—working memory

### Clinical assessments

All clinical assessments were carried out by a neurologist or neuropsychologist. Participants were assessed on the Expanded Disability Status Scale (EDSS) and five cognitive domains using several tests. Information processing speed (IPS) was measured by the verbal Symbol Digit Modalities Test (SDMT) [24]. Verbal memory was measured using the Story Recall Test (SRT) from the Adult Memory and Information Processing Battery (AMIPB) [25] as well as the Recognition Memory Test (RMT) for words [26]. Visual memory was assessed using the RMT for faces [26]. Executive function (EF) was measured by the Stroop Color-Word Interference Test [27] and the Hayling Sentence Completion Test [28]. Working memory was assessed with the Digit Span test, which is an element of the Wechsler Test of Adult Reading (WAIS) [29].

### Image acquisition

MRI scans were acquired using a 3 T Philips Achieva system (Philips Healthcare, Best, The Netherlands) using a 32-channel head-coil. A structural 3D sagittal T1-weighted fast field echo (FFE) scan (repetition time (TR) = 6.9 ms, echo time (TE) = 3.1 ms, 1 mm^3^ isotropic, acquisition time (AT) = 6 min 31 s) and a multi-echo proton density (PD)/T2-weighted scan were obtained (TR = 3500 ms, TE = 19/85 ms, 1 × 1 × 3 mm^3^ anisotropic, AT = 4 min 1 s). A high angular resolution diffusion imaging (HARDI) scan was obtained which consisted of a cardiac-gated spin-echo echo-planar imaging sequence (TR = 24 s [depending on cardiac rate], TE = 68 ms, SENSE factor = 3.1, 2 mm^3^, AT = 33 min 17 s) with 61 isotropically distributed diffusion-weighting directions (*b* = 1200 s/mm^2^) and 7 non-diffusion-weighted volumes (*b* = 0 s/mm^2^). Images were aligned to the anterior commissure (AC) and posterior commissure (PC) line, to reduce the impact of head positioning on image analysis.

### Image preprocessing

**Fig. 1:**
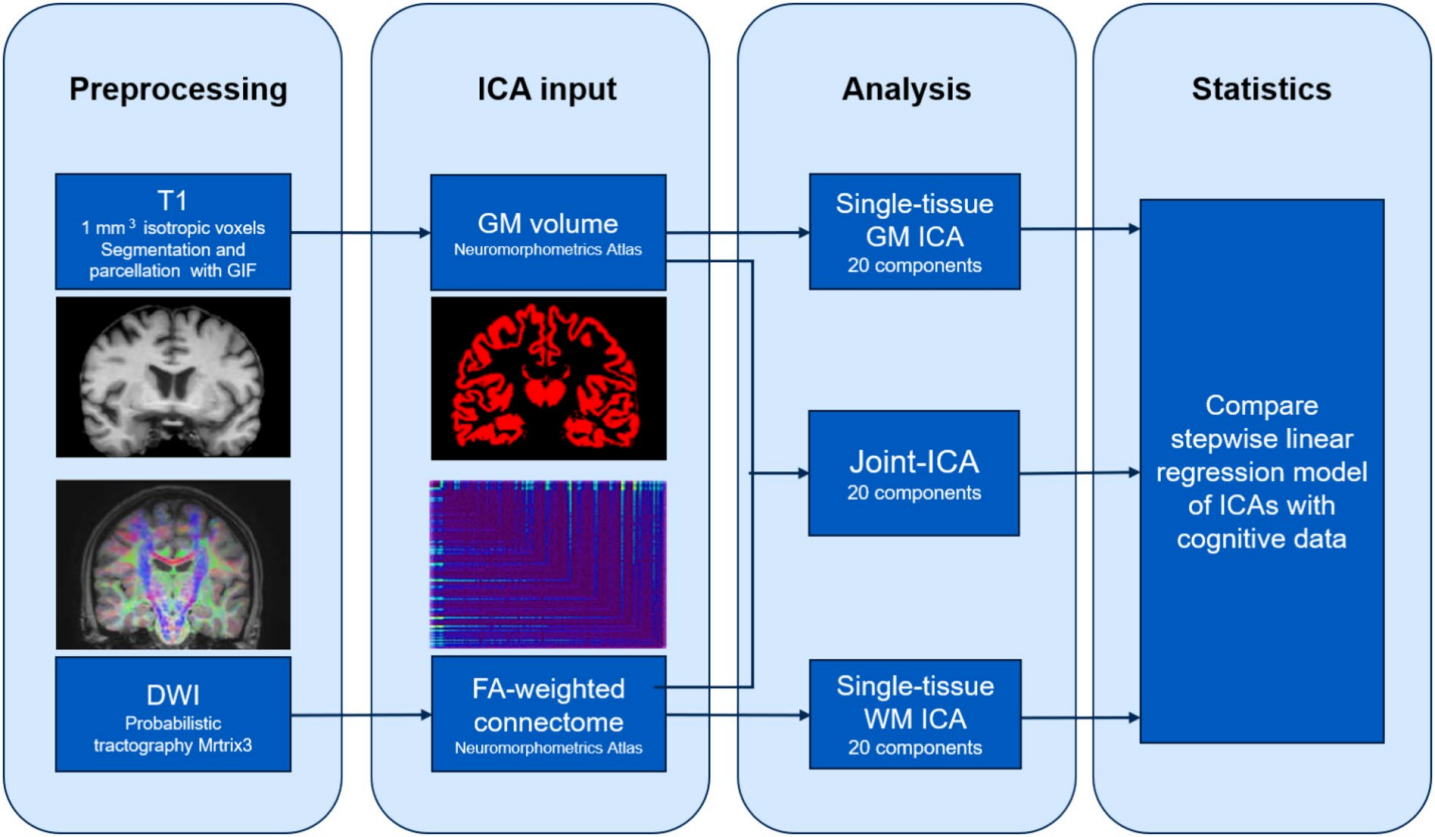
Outline of the image analysis pipelines. Structural T1-weighted scans and DWI were preprocessed and analyzed using GIF and Mrtrix3, respectively, leading to subsequent GM volume and FA-weighted WM connectomes as input for single-tissue and joint-ICA. The ICAs were run using the Matlab-based Fusion ICA Toolbox (FIT: http://trendscenter.org/software/fit) with the Fast-ICA algorithm, resulting in 20 components per ICA model. A stepwise linear regression was run to determine which ICA model showed the highest variance explained in each cognitive domain. *DWI* diffusion-weighted imaging, *FA* fractional anisotropy, *GIF* Geodesic Information Flows, *GM* grey matter, *ICA* independent component analysis, *WM* white matter

Figure 1 shows a schematic overview of the imaging part of the study.

T2 hyperintense lesions were manually outlined by an experienced observer on the PD-weighted images using the semi-automated edge finding tool of JIM v8.0 (Xinapse systems, Aldwincle, UK, http://www.xinapse.com). The lesion masks were then co-registered to the 3D T1-weighted images using a pseudo-T1 image generated by subtracting the PD from the T2-weighted image [30]. Lesion masks were transformed from native space to 3D T1 space using nearest-neighbor interpolation.

Structural T1-weighted images were segmented, after N4 bias field correction [31] and lesion filling [32], using Geodesic Information Flows (GIF) V.3.024 [33]. Lesion filling was performed with the T2 hyperintense lesions in 3D T1 space using a multi-timepoint modality–agnostic patch-based method [32]. GIF, an in-house-developed framework, used the T1-weighted images for segmentation into probabilistic tissue maps and parcellation into regions as defined in the Desikan–Killiany–Tourville (DKT) atlas [34]. Tissue volumes were estimated for cortical GM (CGM), deep GM (DGM), brainstem, WM, cerebral spinal fluid, and the 121 DKT regions (101 representing CGM, 20 representing DGM, see Supplementary Table 1). CGM and DGM were combined for further analysis (FSL v.5.0.9). To adjust for head size, regional volumes were divided by total intracranial volume (TIV) prior to statistical analysis.

For ICA, an established pipeline was used as previously described [14]. In summary, a study-specific GM template was created using the Advanced Normalization Tools (ANTs) software package (https://stnava.github.io/ANTs/) by randomly selecting 44 participants from the cohort. The chosen number was evenly distributed across phenotype groups (RRMS = 15, SPMS = 15, PPMS = 14). The 44 lesion-filled T1-weighted images were registered to MNI152 space by rigid body transformation to create the study-specific template. All participants’ T1-weighted lesion-filled images were then non-rigidly registered to this template. Subsequently, GM probability maps from GIF[33] were transformed to the template space, using the warping matrix previously computed for the T1-weighted images. To account for image deformations after non-linear transformation, we modulated the GM maps by the Jacobian determinants. Finally, 8 mm smoothing kernel was applied to account for inter-subject variability.

### Diffusion imaging processing

The registration between a subject’s 3D T1-weighted image and DWI data was undertaken as described by Mulhert et al. [34]. The 7 non-diffusion-weighted *b* = 0 volumes were averaged to create a mean *b* = 0 image, which was subsequently used to register each of the 61 diffusion-weighted images to. To accurately propagate the GM tissue segmentation from T1 to DW space and account for possible distortions, the 3D T1-weighted image was affine registered to the pseudo-T1 image and the T2-weighted image was first linearly and then nonlinearly registered to the DWI mean *b* = 0 scan. All transformations were concatenated in order to transform the data from native space to DWI space. All registrations were done using the NiftyReg software package (http://niftireg.sf.net).

Processing of the HARDI images was performed as previously described by Charalambous et al. [35]. The images underwent the standard FSL processing pipeline including correction for EDDY current distortion, head motion (FSL v.5.0.9) and echo-planar imaging (EPI) distortions (BrainSuite V.15b). We performed probabilistic tractography in MRtrix3 (V.0.2.14 package), using second-order integration over Fiber Orientation Distributions (iFOD2) which was estimated with constrained spherical deconvolution (CSD). Probabilistic anatomically constrained tractography (ACT) was performed using the 121 GM regions of interest (ROIs) from the DKT atlas, generating 10 million streamlines to construct a tractogram for each subject. Spherical–deconvolution Informed Filtering of Tractograms 2 (SIFT2) was additionally applied to improve fiber accuracy [36]. Fractional anisotropy (FA) maps were generated with dwitensor. White matter connectomes, representing the number of streamlines and mean FA across tracts in the whole brain, were extracted as 121 × 121 matrices based on the GM ROIs from the DKT Atlas.

For ICA input, the number of streamlines and mean FA connectomes for each subject were combined in Matlab to derive a single connectome representing the mean FA weighted by the number of streamlines. WM connectomes were transformed into adjacency matrices for ICA input.

### Independent component analysis

For single tissue as well as joint (GM + WM) ICA, the Fusion ICA Toolbox (FIT: http://trendscenter.org/software/fit) was used. For single-tissue ICA components, parallel-ICA was performed within FIT and the resulting 20 components for each tissue type were examined. For multimodal tissue ICA, the joint-ICA algorithm was utilized with the fast-ICA algorithm to extract 20 components [37–39]. To investigate the stability and statistical reliability of components derived from ICA, ICASSO was performed 20 times [40]. ICASSO is a clustering method, repeating iterations of the ICA several times using random initializations, showing the similarities between iterations of the same independent components. Finally, the iteration closest to the average is used for the outcome, ensuring the generalizability of the method [38].

Next, to determine which brain regions were represented by each component, intensity loadings on GM maps were z-scored and thresholded in Matlab. The image intensities exhibited a range from -10 to 10 and the z-threshold of |z|> 5 was applied to obtain the highest 50% of loadings, resulting in GM component maps. The GM component maps were then overlayed with the DKT atlas, and regions with a minimum overlap of 5% were determined as a significantly contributing ROI of the component.

The WM component vectors were similarly z-scored. The vectors were then transformed back into a matrix connectome, overlayed with the DKT atlas and visually inspected. Due to the high dimensionality of data, a conventional 99% confidence interval threshold was applied to the FA and streamline values. This approach ensured that input data did not contain extreme outliers that could be highly influential in the correlation analyses, while preserving a dynamic range in exploratory analyses. The resulting values were then included as WM connections involved in the component.

## Statistical analysis

All statistical analysis was performed using Rstudio V.2.6.1. The normality of variables was assessed using Shapiro–Wilks tests and by inspection of histograms. Differences between groups in age and sex were evaluated using ANOVA and Chi-square test, respectively. Non-normally distributed variable disease duration and ordinal variable EDSS were compared using Kruskal–Wallis tests and Wilcoxon rank-sum tests post-hoc. Differences in DMT use between groups were evaluated by Chi-square tests and post-hoc Bonferroni correction. Cognitive test scores (uncorrected for age or education, as this study did not seek to determine if cognition was abnormal) were converted into z-scores based on the cohort values, such that negative z-scores represented lower scores relative to the mean performance in the cohort. By converting raw cognitive scores to z-scores, regression coefficients should be more directly comparable across domains. The z-scores were averaged across all tests relating to each cognitive domain, yielding a single score per domain per patient for further analysis. As an exploratory analysis, cognitive domain scores and component loadings between clinical phenotypes (RRMS, PPMS and SPMS) were evaluated using ANCOVA with age and sex as covariates and Tukey HSD post-hoc test when appropriate. Subject component loadings for WM, GM and joint-ICA were transformed into z-scores. Spearman’s correlation coefficients were calculated across subject component loadings and CGM volume, DGM volume, mean FA and number of streamlines across the whole brain, to investigate component relationships with tissue values and establish whether components represented patterns of GM volume or WM microstructural integrity loss. These whole-brain tissue values were used to determine the direction of component associations, as using just the ROIs within each component would have likely resulted in smaller correlations. However, in each component, volumes of individual GM ROIs and the number of streamlines and FA in individual WM connections were also correlated to the loading factors, to determine the directionality of individual corresponding component regions.

Partial correlation was performed between cognitive scores and component loadings, with age and sex as covariates, to determine the univariate association of the joint-ICA components with cognitive domains. Pearson’s partial correlation coefficient was calculated for all cognitive domains. A data-driven stepwise linear regression model was calculated for joint-ICA components, with age and sex as covariates, to evaluate which joint-ICA components would best explain each cognitive domain. Finally, to determine whether joint-ICA components increased the variance explained compared to single-tissue ICA, a stepwise linear regression for the single-tissue ICAs was performed. The joint-ICA and single-tissue ICA models were compared using a likelihood ratio test. A p < 0.05 was considered statistically significant. Statistical results were corrected for multiple comparisons with false discovery rate (FDR) unless mentioned otherwise.

## Results

### People with SPMS have worse clinical and IPS scores compared to people with RRMS

Exploratory analysis was performed on demographic, clinical and cognitive characteristics for the RRMS (*n* = 53), SPMS (*n* = 22) and PPMS (*n* = 14) groups (Table 1). Sex did not significantly differ between groups. A one-way ANOVA showed group differences for age (F(2,86) = 15.03, *p* < 0.001), with post-hoc tests revealing that people with SPMS and PPMS were older compared to RRMS (*p* < 0.001 and *p* < 0.01, respectively). Kruskal–Wallis tests revealed disease duration (H(2) = 26.08, *p* < 0.001) and EDSS (H(2) = 9.59, *p* < 0.001) significantly differed between groups, with the SPMS group having longer disease duration and higher EDSS compared to RRMS (*p* < 0.001 and *p* < 0.001, respectively) and PPMS (*p* < 0.001 and *p* < 0.05, respectively). People with PPMS also had higher EDSS scores compared to people with RRMS (*p* < 0.01).

For measures of cognition, ANCOVA tests, adjusting for age and sex, revealed a significant main effect of group for IPS (F(2,84) = 6.66, *p* < 0.01) and visual memory (F(2,84) = 4.16, *p* < 0.05) with post-hoc tests revealing that this was driven by significantly lower scores in individuals with SPMS compared to those with RRMS (*p* < 0.01 and *p* < 0.05, respectively). There were no significant differences between scores in other cognitive domains between groups.

### Joint-ICA components reflect biological networks and uncover patterns of GM and WM features that are not necessarily apparent in either tissue alone

**Fig. 2:**
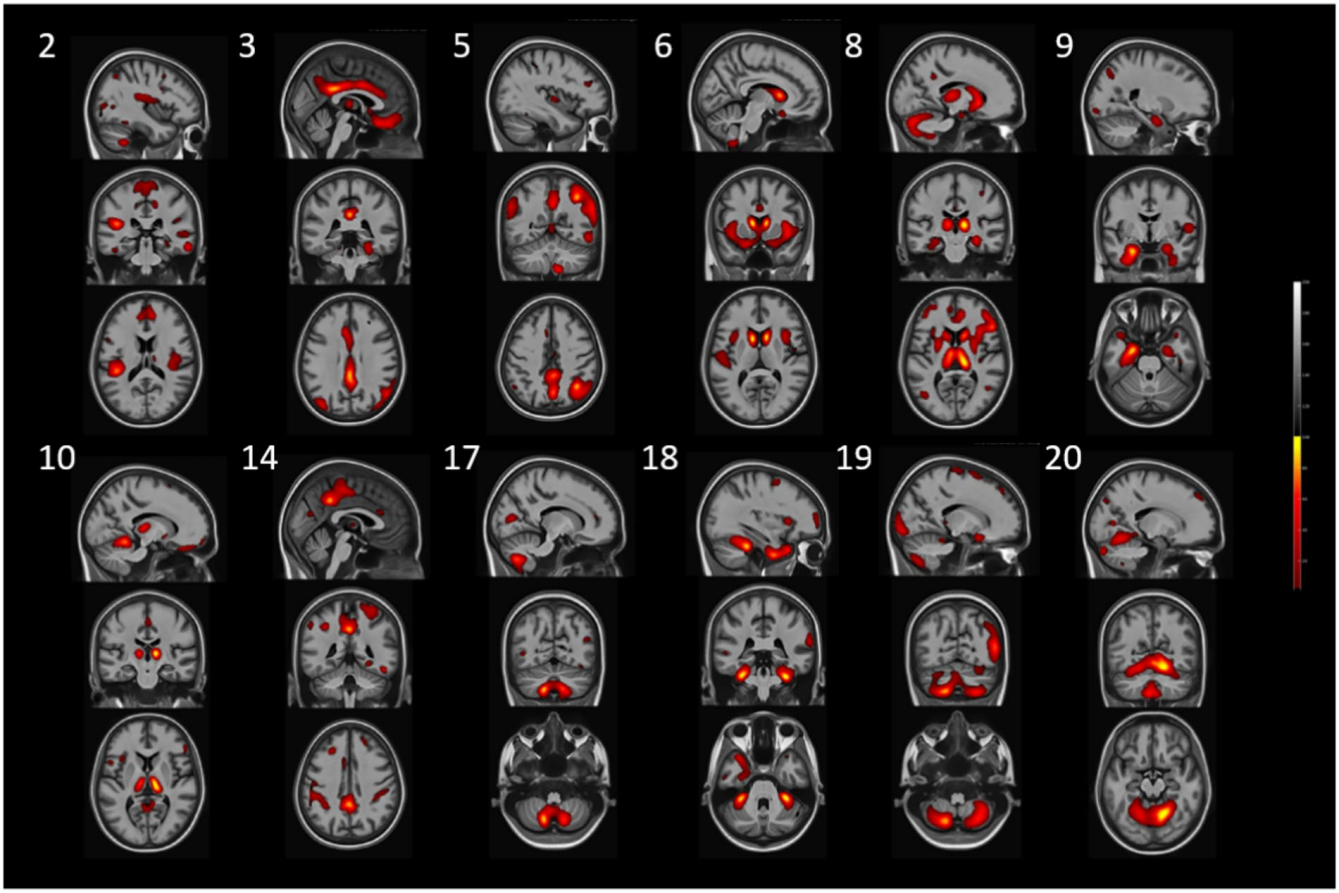
Grey matter elements of the joint-ICA components. Components that associated with cognitive domains in either univariate or multivariate analysis are displayed here. Component 2 is a parietal–cerebellar component, involving mainly cerebellar lobules VIII–X, posterior cingulate, precuneus and parietal operculum. A cingulate–frontal pattern is displayed by component 3, encompassing mainly the posterior and middle cingulate, medial frontal cortex and gyrus rectus. Component 5 is a posterior parietal–occipital pattern, spanning mainly the including posterior cingulate, angular gyrus, precuneus, lingual gyrus and amygdala. Component 6 resembles the default-mode network (precuneus, posterior cingulate and orbital gyrus) and salience network (anterior insula and anterior cingulate). A deep grey matter pattern is shown by component 8, encompassing mainly the thalamus, amygdala, putamen and caudate nucleus. Component 9 is a left temporal–cerebellar pattern, representing the cerebellar exterior and the left entorhinal area, hippocampus and para-hippocampal gyrus. Component 10 is a visual-like network, involving the calcarine cortex, occipital fusiform gyrus and thalamus. A cerebellar–inferior occipital pattern was shown by component 14, encompassing the cerebellum, occipital fusiform gyrus and inferior occipital gyrus. Component 17 is a cerebellar–temporal–cingulate pattern, involving mainly the cerebellum, transverse temporal gyrus and posterior cingulate. Component 18 is an opercular–occipital component (frontal operculum, opercular part of the inferior frontal gyrus, cuneus and calcarine cortex). Occipital–frontal component 19 involves mainly the occipital pole, calcarine cortex and subcallosal area. Component 20 is a cerebellar–temporal–occipital pattern, encompassing the cerebellum, lingual gyrus and middle temporal gyrus. *ICA* independent component analysis

**Fig. 3:**
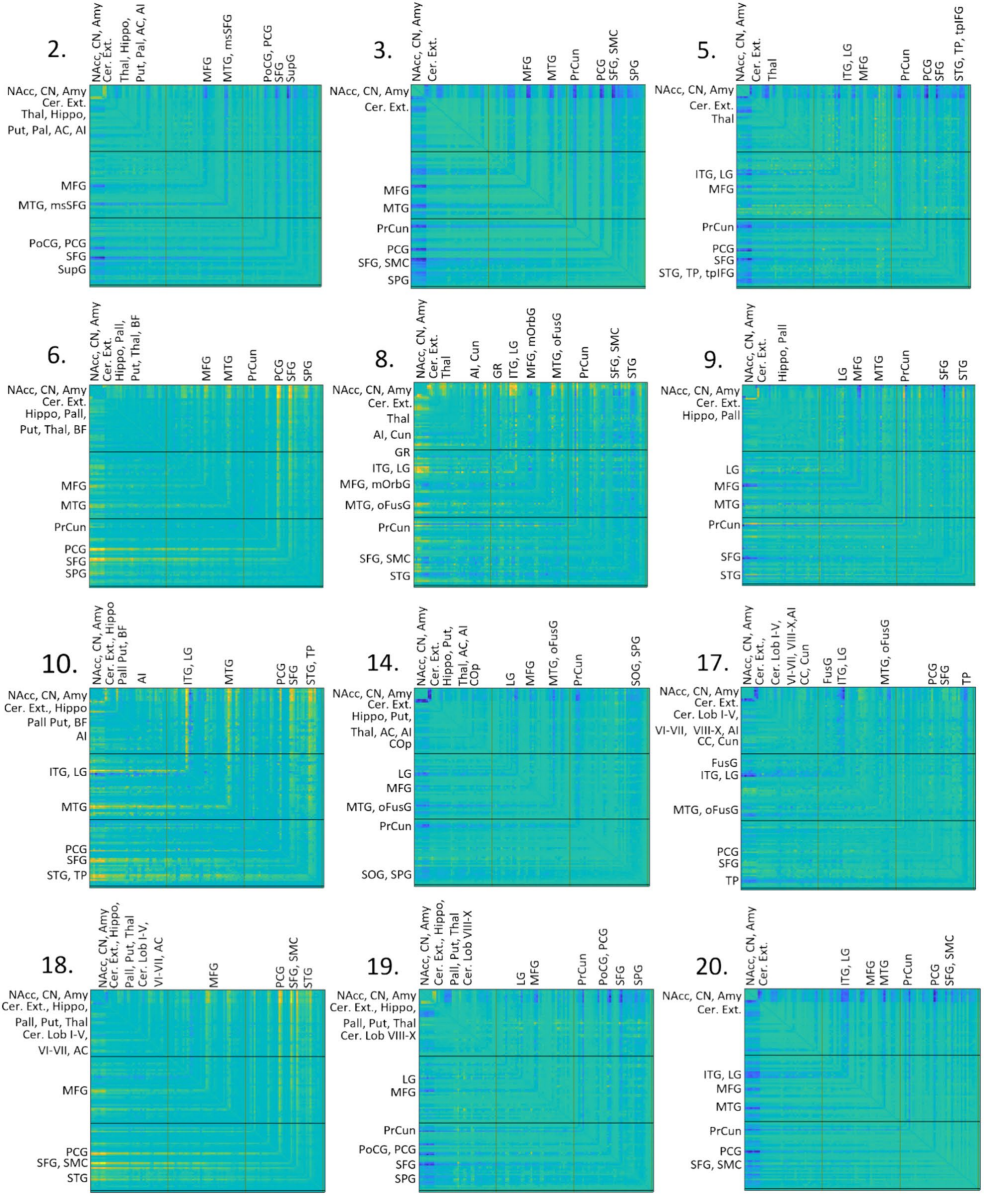
WM elements of the joint-ICA components. Components that associated with cognitive domains in either univariate or multivariate analysis are displayed here. WM connectomes are shown with the node-to-node connections within the 99% confidence interval labeled. Component 2 involves predominantly connections between the limbic and DGM regions to frontal and parietal structures. For component 3 WM connections between amygdala, NAcc, CN and CerExt and precuneus and frontal regions showed highest loadings. The highest loadings of component 5 were on connections between amygdala, NAcc, CN and CerExt to frontal regions, thalamus and precuneus. Component 6 shows the highest loading WM connections connecting several DGM and cerebellar structures with specifically the precentral and superior frontal gyrus. WM connections of component 8 with high loadings came from the amygdala, striatum and cerebellum to temporal regions, parietal and occipital regions. Component 9 shows the strongest connections between the amygdala, NAcc, CN and CerExt, precuneus, frontal and temporal regions. A WM pattern between the DGM structures, cerebellum, temporal regions, SFG and lingual gyrus was shown by component 10. Component 14 showed high-loading connections between amygdala, NAcc, CN and cerebellum, lingual gyrus, parietal and temporal regions. Component 17 showed a pattern of connections present between the amygdala, NAcc, CN and cerebellum, temporal lobe structures, lingual and occipital fusiform gyrus. Component 18 involved mainly connections between several DGM structures, the cerebellum and frontal regions and the supplementary motor cortex. The WM pattern of component 19 shows connections between DGM, cerebellum, frontal and parietal regions. Component 20 involves the highest connections between the amygdala, NAcc, CN and CerExt, precuneus, lingual gyrus and temporal regions. *AC* anterior cingulate, *AI* anterior insula, *Amy* Amygdala, *aOrbG* anterior orbital gyrus, *BF* basal forebrain, *CC* calcarine cortex, *CerExt* cerebellum exterior, *CerLob* cerebellar lobules, *CN* caudate nucleus, *COp* central operculum, *Cun* cuneus, *FOp* frontal operculum, *FusG* fusiform gyrus, *Hippo* hippocampus, *GR* gyrus rectus, *ICA* independent component analysis, *IOG* inferior occipital gyrus, *ITG* inferior temporal gyrus, *LG* lingual gyrus, *MC* middle cingulate, *MFG* middle frontal gyrus, *mOrbG* medial orbital gyrus, *msSFG* medial segment of the superior frontal gyrus, *MTG* middle temporal gyrus, *NAcc* nucleus accumbens, *oFusG* occipital fusiform gyrus, *OP* occipital pole, *opIFG* opercular part of the inferior frontal gyrus, *OrbG* orbital gyrus, *Pall* pallidum, *PCG* precentral gyrus, *PoCG* postcentral gyrus, *POp* parietal operculum, *PrCun* precuneus, *Put* putamen, *SCA* subcallocal area, *SFG* superior frontal gyrus, *SMC* supplementary motor cortex, *SOG* superior occipital gyrus, *SPG* superior parietal gyrus, *STG* superior temporal gyrus, *SupG* supramarginal gyrus, *Thal* thalamus, *TP* temporal pole, *tpIFG* triangular part of the inferior frontal gyrus, *TTG* transverse temporal gyrus, *WM* white matter

Twenty ICA components were derived from the joint-ICA. Figures 2 and 3 illustrate the pattern of GM and WM contribution to joint-ICA components associated with cognitive data, respectively. For a full illustration of all components, please refer to Supplementary Figs. 1 and 2. A further detailed description of the components is provided in Table 2. For components 1, 3, 4, 5, 8, 11, 15, and 20, patterns of GM and WM showed alignment, such that the GM regions associated with each component were complimented by the presence of WM streamlines that connected with these regions, although usually the WM element included more regions. For example, component 3 showed a cingulate–frontal pattern, including the frontal medial cortex, posterior and middle cingulate, gyrus rectus, fusiform gyrus, middle occipital gyrus, lingual gyrus and inferior temporal gyrus, and WM contributions including tracts that connect between them. In contrast, components 2, 6, 7, 9, 10, 12, 13, 14, 16, 17, 18 and 19 reflected combinations of patterns of GM and WM that did not spatially align, such that WM tracts involved in the component did not connect to most of the GM regions also associated in these components. For example, the GM regions of component 6 resembled the default-mode network (DMN) (precuneus, posterior cingulate, angular gyrus, inferior medial frontal) and salience network (SN) (anterior cingulate, insula). However, the highest loading WM tracts specifically connected several DGM regions (nucleus accumbens, caudate nucleus and amygdala) and cerebellar exterior with frontal regions (precentral gyrus and superior frontal gyrus). A detailed description of the other components is provided in Table 2.

**Table 2:** List of clinically relevant joint-ICA components with their corresponding GM ROIs and top 0.1% WM connections

	GM ROIs	WM connections		
Comp2	Precuneus↑, posterior cingulate↑, cerebellar exterior↑, cerebellar lobules VIII–X↑, transverse temporal, postcentral gyrus medial segment↓, medial frontal cortex↑, parietal operculum↓, planum temporale↑, and anterior cingulate↑	Left nucleus accumbens	–	Right superior frontal gyrus↑
		Right amygdala	–	Right superior frontal gyrus↑
		Left amygdala	–	Right superior frontal gyrus↑
		Right caudate nucleus	–	Right superior frontal gyrus↑
		Left caudate nucleus	–	Right superior frontal gyrus↑
		Right cerebellar exterior	–	Right superior frontal gyrus↑
		Left cerebellar exterior	–	Left middle frontal gyrus↑
		Left cerebellar exterior	–	Right superior frontal gyrus↑
Comp3	Frontal medial cortex↓, posterior↑ and middle cingulate↑, putamen↓, pallidum↑, thalamus↑, middle temporal gyrus↓, occipital fusiform gyrus↓, gyrus rectus↑, fusiform gyrus↑, middle occipital gyrus↓, lingual gyrus↓ and inferior temporal gyrus↑	Left amygdala	–	Right precentral gyrus↓
		Left amygdala	–	Right superior frontal gyrus↓
		Left caudate	–	Right precentral gyrus↓
		Left caudate	–	Right superior frontal gyrus↓
		Right cerebellar exterior	–	Right precentral gyrus↓
		Right cerebellar exterior	–	Right superior frontal gyrus↓
		Left cerebellar exterior	–	Right precentral gyrus↓
		Left cerebellar exterior	–	Right superior frontal gyrus↓
Comp5	Posterior cingulate↓, angular gyrus↑, precuneus↓, hippocampus↓, amygdala↓, parietal superior gyrus↓, lingual gyrus↓, fusiform gyrus↑, cerebellar exterior↓, anterior cingulate↓, medial orbital gyrus↓, superior temporal gyrus↓, parahippocampal gyrus↓, subcallosal area↓, calcarine cortex↓, middle↓ and superior occipital gyrus↓, posterior orbital gyrus ↓	Right nucleus accumbens	–	Left precuneus↑
		Right nucleus accumbens	–	Left precentral gyrus↑
		Right nucleus accumbens	–	Right superior frontal gyrus↑
		Left caudate nucleus	–	Left precentral gyrus↑
		Right cerebellar exterior	–	Left precentral gyrus↑
		Right cerebellar exterior	–	Right superior frontal gyrus↓
		Left cerebellar exterior	–	Left precentral gyrus↑
		Left cerebellar exterior	–	Right superior frontal gyrus↓
Comp6	Caudate nucleus↓, precuneus↓, posterior cingulate↓, anterior insula↓, anterior cingulate↓, posterior↓, medial↓ and lateral↓ orbital gyrus, frontal operculum↓, inferior frontal orbital gyrus↑, hippocampus↓ and middle occipital gyrus↓	Right nucleus accumbens	–	Right precentral gyrus↑
		Left nucleus accumbens	–	Right precentral gyrus↑
		Right amygdala	–	Right precentral gyrus↑
		Right amygdala	–	Right superior frontal gyrus↑
		Left amygdala	–	Right precentral gyrus↑
		Left amygdala	–	Right superior frontal gyrus↑
		Right cerebellar exterior	–	Right precentral gyrus↑
		Left cerebellar exterior	–	Right precentral gyrus↑
Comp8	Thalamus↓, caudate nucleus↓, putamen↓, amygdala↓, nucleus accumbens↓, hippocampus↓, anterior insula↓, cerebellum lobules VIII-X↓, frontal operculum↓, triangular part inferior frontal gyrus↓, anterior↓ and posterior cingulate↓ and pallidum↓	Right amygdala	–	Left cerebellar exterior↓
		Right amygdala	–	Left cuneus↓
		Right amygdala	–	Right inferior temporal gyrus↓
		Right amygdala	–	Left lingual gyrus↓
		Right amygdala	–	Left precuneus↓
		Left amygdala	–	Left precuneus↓
		Right caudate nucleus	–	Left lingual gyrus↓
		Right anterior insula	–	Left lingual gyrus↓
Comp9	Left entorhinal area↓, left parahippocampal gyrus↓, amygdala↓, hippocampus↓, middle temporal gyrus↓, precentral gyrus medial segment↓, cerebellum exterior↓, caudate nucleus↓, temporal pole↓ and fusiform gyrus↓	Right nucleus accumbens	–	Left middle frontal gyrus↓
		Right nucleus accumbens	–	Left precuneus↓
		Right nucleus accumbens	–	Left precentral gyrus↓
		Right nucleus accumbens	–	Left superior frontal gyrus↓
		Left nucleus accumbens	–	Left superior frontal gyrus↓
		Right amygdala	–	Left middle frontal gyrus↓
		Right amygdala	–	Left superior frontal gyrus↓
		Left amygdala	–	Left superior frontal gyrus↓
Comp10	Calcarine cortex↓, occipital fusiform gyrus↓, thalamus↓, lingual gyrus↓, cuneus↓, precuneus↓, inferior occipital gyrus↓, cerebellar lobules I–V↓, planum temporale↓, transverse↓ and superior temporal gyrus↓, gyrus rectus↓ and posterior cingulate↓	Right nucleus accumbens	–	Right inferior temporal gyrus↑
		Right amygdala	–	Right inferior temporal gyrus↑
		Right amygdala	–	Right middle temporal gyrus↑
		Left amygdala	–	Right inferior temporal gyrus↑
		Left amygdala	–	Right lingual gyrus↓
		Left caudate nucleus	–	Right inferior temporal gyrus↑
		Right cerebellar exterior	–	Right inferior temporal gyrus↑
		Left cerebellar exterior	–	Right inferior temporal gyrus↑
Comp14	Occipital fusiform gyrus↓, cerebellar vermal lobules I–V↓and VIII–X↓, posterior cingulate↓, inferior occipital gyrus↑, medial segment precentral gyrus↓, lingual gyrus↑ and cerebellar exterior↓	Left nucleus accumbens	–	Left cerebellar exterior↓
		Right amygdala	–	Right cerebellar exterior↓
		Left amygdala	–	Right cerebellar exterior↓
		Right caudate nucleus	–	Right cerebellar exterior↓
		Right caudate nucleus	–	Left cerebellar exterior↓
		Left caudate nucleus	–	Right cerebellar exterior↓
		Left caudate nucleus	–	Left cerebellar exterior↓
		Right cerebellar exterior	–	Left cerebellar exterior↓
Comp17	Cerebellar lobules I–V ↓, VIII–X↓, VI–VII↓, cerebellar exterior↓, posterior cingulate↓, temporal transverse gyrus↓, postcentral gyrus↓, middle cingulate↓, orbital part inferior frontal gyrus↓, central operculum↓, supramarginal gyrus↓ and planum temporale↓	Right nucleus accumbens	–	Left lingual gyrus↓
		Right amygdala	–	Right cerebellar exterior↓
		Right amygdala	–	Left lingual gyrus↓
		Right caudate nucleus	–	Right cerebellar exterior↓
		Left caudate nucleus	–	Right cerebellar exterior↓
		Left caudate nucleus	–	Left lingual gyrus↓
		Right cerebellar exterior	–	Left cerebellar exterior↓
		Right cerebellar exterior	–	Left lingual gyrus↓
Comp18	Cuneus↓, frontal operculum↓, opercular part inferior frontal gyrus↓, gyrus rectus↓, precuneus↓, fusiform gyrus↓, left entorhinal area↓, temporal pole↓, superior occipital gyrus↓, medial frontal cortex↓ and calcarine cortex↓	Left nucleus accumbens	–	Right superior frontal gyrus↓
		Right amygdala	–	Right superior frontal gyrus↓
		Left amygdala	–	Right superior frontal gyrus↓
		Left caudate nucleus	–	Right precentral gyrus↓
		Left caudate nucleus	–	Right superior frontal gyrus↓
		Right cerebellar exterior	–	Right precentral gyrus↓
		Right cerebellar exterior	–	Right superior frontal gyrus↓
		Left cerebellar exterior	–	Right superior frontal gyrus↓
Comp19	Calcarine cortex↓, occipital pole↓, inferior↓ and middle occipital gyrus↓, lingual gyrus↑, subcallosal area↑, caudate nucleus↑, inferior↑ and middle temporal gyrus↑, cerebellar exterior↑, medial orbital gyrus↑ and occipital fusiform gyrus↓	Right nucleus accumbens	–	Left superior frontal gyrus↓
		Left amygdala	–	Left superior frontal gyrus↓
		Right caudate nucleus	–	Left precentral gyrus↓
		Right caudate nucleus	–	Left superior frontal gyrus↓
		Right cerebellar exterior	–	Left precentral gyrus↓
		Right cerebellar exterior	–	Left superior frontal gyrus↓
		Left cerebellar exterior	–	Left precentral gyrus↓
		Left cerebellar exterior	–	Left superior frontal gyrus↓
Comp20	Cerebellar lobules I–V↑, VI–VII↑, VIII–X↑, cerebellar exterior↑, lingual gyrus↑, inferior↑ and middle occipital↑, inferior↑ and middle temporal gyrus↑, opercular part inferior frontal gyrus↑, anterior cingulate↑ and fusiform gyrus↑	Left nucleus accumbens	–	Right precentral gyrus↓
		Right amygdala	–	Left precuneus↓
		Right amygdala	–	Right precentral gyrus↓
		Left amygdala	–	Right middle temporal gyrus↓
		Left amygdala	–	Right precentral gyrus↓
		Left caudate nucleus	–	Right precentral gyrus↓
		Right cerebellar exterior	–	Right precentral gyrus↓
		Left cerebellar exterior	–	Right precentral gyrus↓

Components that associated with cognitive domains in either univariate or multivariate analysis are displayed here. Parcellation masks were overlayed with the GM part of components, and the connectomes were labelled to identify the regions and connections in each component. Volumes of GM regions and the number of streamlines and FA for WM connections were correlated with component loadings to identify the directionality of the tissue values of regions (↑ positive correlation or ↓ negative correlation). GM regions are described bilaterally unless mentioned otherwise. *Comp* component, *FA* fractional anisotropy, *GM* grey matter, *ICA* independent component analysis, *ROI* region of interest, *WM* white matter

Joint-ICA components were visually compared to single-tissue GM and WM components. In GM, 11 of the 20 joint-ICA components were similar to single-tissue GM (2, 3, 6, 7, 8, 10, 11, 13, 14, 16 and 17), although the components usually encompassed more GM regions. For WM, joint-ICA components (6, 7, 8, 10, 14, 16, 17 and 18) showed similar patterns to single-tissue WM components. Comparable to the GM part, the WM part of joint-ICA components generally showed a more widespread distribution of high-loading regions, compared to single-tissue WM ICA. In combination, six joint-ICA components overlapped with both single-tissue GM and WM components (6, 7, 8, 10, 16 and 17).

Exploring the differences in components between MS phenotypes using ANCOVA tests adjusted for age and sex, a significant difference in loadings was found for components 4 (F(2,84) = 4.73, *p* < 0.05) and 20 (F(2,84) = 5.68, *p* < 0.01). A strong trend in difference between phenotypes was observed for component 6, but did not reach statistical significance (F(2,84) = 2.98, *p* = 0.05) (Supplemental Table S3). Post-hoc tests revealed that people with SPMS had significant higher loadings on component 4 compared to PPMS (*p* < 0.05) and showed a trend in higher loadings compared to RRMS (*p* = 0.05). People with PPMS had higher loadings on component 20 compared to RRMS (*p* < 0.01) and SPMS (*p* < 0.05). Exploring the strong trend in component 6, post-hoc tests showed that people with SPMS had generally higher loadings on component 6 compared to people with RRMS, but this did not reach statistical significance (*p* = 0.05).

### Joint-ICA components show significant associations with GM volume but, after allowing for FDR, not with WM measures

Of the 20 components, 12 showed significant associations with either GM volumes or WM structural integrity. For simplicity, only statistically significant results are presented in color in Table 3. For all correlations, see Supplementary Table S4.

**Table 3:**
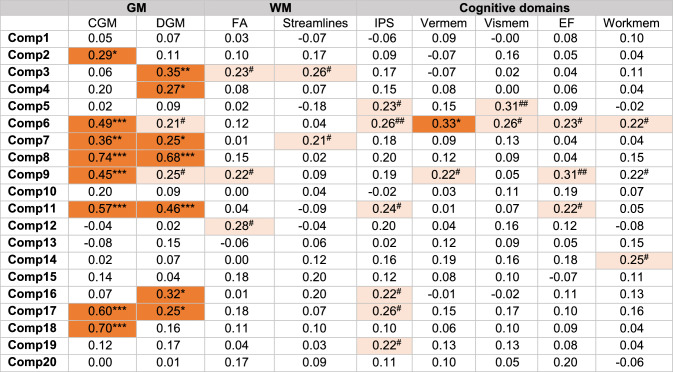
Correlation between joint-ICA component loadings, tissue volumes and cognitive domains

To determine the association between joint-ICA components and GM and WM tissue values (CGM volume, DGM volume, mean FA and number of streamlines across the whole brain) Spearman’s correlation coefficients were calculated. For the association between joint-ICA components and the cognitive domains, a partial correlation was performed with age and sex as covariates. Orange and red indicate significant negative correlations before and after FDR correction, respectively. ^#^*p* < 0.05 before FDR-correction, ^##^*p* < 0.01 before FDR-correction, *p < 0.05, **p < 0.01, ***p < 0.001 after FDR correction. *CGM* cortical grey matter, *Comp* component, *DGM* deep grey matter, *EF* executive function, *FA* fractional anisotropy, *GM* grey matter, *ICA* independent component analysis, *IPS* information processing speed, vermem—verbal memory, vismem—visual memory, workmem—working memory, *WM* white matter

Higher loadings on components 7 (*r* = 0.36, *p* < 0.01; *r* = 0.25, *p* < 0.05), 8 (*r* = 0.74, *p* < 0.001; *r* = 0.68, *p* < 0.001), 11 (*r* = 0.57, *p* < 0.01; *r* = 0.46, *p* < 0.001), 17 (*r* = 0.60, *p* < 0.001; *r* = 0.25, *p* < 0.05) were associated with both higher CGM and DGM volumes, respectively. Components 3 (*r* = 0.35, *p* < 0.01), 4 (*r* = 0.27, *p* < 0.05) and 16 (*r* = 0.32, *p* < 0.05) correlated only with higher DGM volumes. Components 2 (*r* = 0.29, *p* < 0.05), 6 (*r* = 0.49, *p* < 0.001), 9 (*r* = 0.54, *p* < 0.001) and 18 (*r* = 0.70, *p* < 0.001) only showed association with higher CGM volumes, their regions being predominantly CGM distributed.

Associations between FA values and component loadings were found for components 3 (*r* = 0.23, *p* < 0.05), 9 (*r* = 0.22, *p* < 0.05) and 12 (*r* = 0.29, *p* < 0.01), and between the number of streamlines and subject loadings for components 3 (*r* = 0.26, *p* < 0.05) and 7 (*r* = 0.21, *p* < 0.05. However, these findings did not remain significant after FDR correction.

Which GM ROIs and WM connections in each component were individually associated with tissue values is shown in Table 2.

### Joint-ICA components show significant associations with cognitive measures

Univariate analyses of joint-ICA components, after allowing for age and sex differences, are shown in Tables 3 and S3. Component 6 significantly correlated with verbal memory (*r* = − 0.33, *p* < 0.05) after FDR correction, while components 5 and 9 showed trends in association with visual memory (*r* = − 0.31, *p* = 0.05) and EF (*r* = − 0.31, *p* = 0.06), respectively.

Stepwise linear regression assessing multivariate associations of both single-tissue ICA and joint-ICA with each cognitive domain, with age and sex as covariates, are shown in Table 4. Stepwise linear regression from both single-tissue and joint-tissue ICA were compared, showing that for some, but not all cognitive domains, joint-ICA better explained outcomes than either GM or WM single-tissue ICA (Table 4). Visual memory (Adjusted R^2^ (Adj.R^2^) = 0.30) and EF (Adj.R^2^ = 0.35) were best explained by joint-ICA compared to GM ICA (*p* < 0.05, *p* < 0.01, respectively) and WM ICA (*p* < 0.001, *p* < 0.01, respectively). Verbal memory showed the highest variance explained using joint-ICA (adjusted R2 (Adj.R^2^) = 0.24), but differences with GM and WM ICA were not significant. For working memory, WM ICA (Adj.R^2^ = 0.23) explained the most variance and was significantly higher compared to joint-ICA (*p* < 0.01) and GM ICA (*p* < 0.01). For IPS the highest variance was explained using the single-ICA GM model (Adj.R^2^ = 0.32), although this difference was not significant when compared to joint- and WM ICA.

**Table 4:**
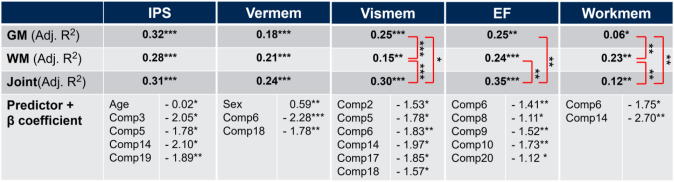
Stepwise linear regression models of joint-ICA and single-tissue ICA components as predictors of cognitive performance

Stepwise linear regression models were performed to determine the variance explained by joint-ICA and single-tissue ICA components for each cognitive domain. The significant contributing components for the joint-ICA model are shown as predictors. The performance of joint-ICA and single-tissue ICA models against each other was compared using a likelihood ratio test (significant differences between models are highlighted by the red brackets). *p* < 0.05*, *p* < 0.01**, *p* < 0.001***. Adj.R^2^—adjusted R^2^, Comp—component, *EF* executive function, *GM* grey matter, *ICA* independent component analysis, *IPS* information processing speed, vermem—verbal memory, vismem—visual memory, *WM* white matter, workmem—working memory

## Discussion

In this study, we investigated whether combining measures of GM and WM using a joint-ICA approach would extract novel patterns when compared with single-tissue ICA, and better explain cognitive function in MS. We found that joint-ICA identified networks of GM and WM features beyond those captured by single-tissue ICA, and outperformed single-tissue ICA in explaining cognition in two of the five domains assessed (for IPS and verbal memory no model significantly outperformed one another, while for working memory single-tissue WM ICA did). These findings show that features of GM and WM both contribute to explaining cognitive function, but show that their combined effect can be greater than either alone, depending on the cognitive domain.

A key aim of this study was to determine whether covarying patterns of GM and WM changes in MS could be derived using joint-ICA and whether these reflected known functional brain networks. Of the components derived using joint-ICA, several appeared to correspond with functional networks such as the DMN and SN (component 6), a cortico-basal ganglia network (component 8), cerebellar networks (components 11 and 20) and visual network (component 19). This supports previous findings that show that GM and WM pathologies can be interrelated [9], and so suggesting mediation through neural networks. However, not all joint or single-tissue ICA patterns did so, and it should be noted that the direct propagation of changes through neural networks is not the only explanation for co-varying features [41]. In particular, for ICA patterns confined to or heavily weighted towards GM or WM other processes may be more relevant, such as regionally targeted pathology, differential regional vulnerability to a pathological process [41], or possible sequential regional pathology [42].

Previous studies using joint-ICA to assess structural MRI and DWI in dementia, obsessive–compulsive disorder and bipolar disorder have used TBSS, limiting the search for covarying patterns to large WM tracts such as the inferior and superior longitudinal fasciculus and corticospinal tracts, which will be associated with equivalently large GM regions [19–21]. In the present work, the joint-ICA was given free rein to include any GM or WM regions, and the top node-to-node connections within the 99% confidence interval were used as strongest contributing to the component. For some components, the WM tracts did not show alignment with the GM regions, i.e., the WM streamlines contributing to a joint-ICA component were not directly connected with the GM element of the component. Interestingly, some patterns of WM connections were present in multiple joint-ICA components, e.g., those between DGM structures, cerebellar exterior and cortical regions like the precuneus, middle and superior frontal gyrus, precentral gyrus and lingual gyrus. This suggests that these patterns of WM changes are relevant to multiple networks. Several of the cortical nodes associated with these connections, such as the precuneus, superior frontal gyrus and lingual gyrus, are known hubs in the brain [43]. Hub alterations are present from early in the disease and can eventually become functionally overloaded. This can affect multiple functional and structural networks [44], which is possibly why connections between them feature in several joint-ICA components.

Turning to associations between ICA patterns and cognitive outcomes, we found that for three domains joint-ICA increased the variance explained in a stepwise linear regression model compared to single-tissue ICA (visual memory, EF and verbal memory, although for the latter the difference was not significant). We anticipated that joint-ICA components would be more sensitive to patterns of change compared to single-tissue ICA, and that, as these patterns span both GM and WM, which are both integral in supporting network function and cognition [14, 16], they would be better able to explain cognitive function. However, our hypothesis was not confirmed with working memory, whereby single-tissue ICA of WM was significantly better able to explain outcomes. Working memory consists of multiple stages, and shows a distributed coalition of regions being recruited across the brain; it is plausible that it is the stability and efficiency of connections, rather than GM changes, that has a dominant effect on impairments in MS and is most relevant to this function [45, 46]. When considered together, these findings suggest that for some cognitive functions, changes in one tissue may be more relevant than in another, and that in such instances, joint-ICA components simply dilute findings in the most relevant tissue. It also highlights that single-tissue and joint ICA are complimentary, rather than joint-ICA simply being a more sensitive alternative to single-tissue ICA. This may also help to explain the varied results in previous research that has sought to capture heterogeneity in cognitive domains using either WM integrity or GM volume alone [10, 14, 16].

In our analysis, we found that twelve components were associated with cognitive function, either independently or when combined with other components in multivariate regression analyses. Of note, replicating previous ICA MRI observations [14], not all components were linked with cognitive function. This reminds us that neurological and cognitive functions are served by specific neural networks, and whilst these are themselves linked, when seeking to explain clinical outcomes their contribution may diluted, or entirely overlooked, in whole-brain measures. Of the twelve components that did associate with cognition, the majority demonstrated this with only one cognitive domain, although several showed associations across multiple cognitive domains. For example, component 5, which represents a posterior parietal–occipital pattern, was associated with both IPS and visual memory. This component includes classical network hubs such as the precuneus, lingual gyrus and superior parietal gyrus, and such network hubs are implicated in several higher-order cognitive processes, and are fundamental in integrating neural systems in the brain [43]. Given this, it is unsurprising that this component was associated with multiple cognitive domains. Similarly, for component 14, which included cerebellar–inferior occipital regions, significant associations to visual and working memory were observed. Notably, this component included the posterior cingulate cortex, a key network hub that is implicated in both cognitive processes, and is likely influential to both their functioning [47]. For verbal and visual memory, opercular–occipital component 18 predicted the outcomes most strongly. The association with verbal memory can be attributed to the inferior frontal pars opercularis, a region that is fundamental in auditory and speech processing. The association with visual memory is exhibited by the involvement of the frontal operculum, a region implicated in cognitive control, and several parieto-occipital regions predominantly linked to visuospatial functions [48]. The integration of both the frontal operculum and parieto-occipital regions within this component supports existing evidence that the frontal operculum’s cognitive control function particularly applies in task-dependent activity in posterior networks [48]. Of note, component 6, which closely aligned to the DMN and SN networks, was associated with scores on all cognitive domains, either in univariate or multivariate analysis. This association with all cognitive domains aligns with previous research demonstrating that the DMN is associated with global information integration for conscious processing and contributes to task performance [49]. A similar DMN component relating to several cognitive scores in previous ICA studies [14] further supports this.

When considering the components that are exclusively related to one cognitive domain, the patterns of associated GM and WM were biologically plausible. For example, components involved in visual memory mainly represented parietal, occipital, and cerebellar areas that are widely implicated in visual memory or visuospatial function, including the precuneus [50], lingual gyrus [51], superior parietal gyrus [52] and occipital fusiform gyrus [53]. Interestingly, the posterior cerebellum, especially lobules VIII–X, was frequently represented in components associated with visual memory. Cerebellar atrophy can present early on in MS [54], and GM changes in the posterior cerebellum specifically affect cognitive symptoms [55], including visuospatial functions [56, 57], possibly reflecting this in our components. Finally, higher scores on the EF domain tests were associated with components that showed high node-to-node WM connections between the DGM/cerebellum and the frontal lobe (especially the middle and superior frontal gyrus), aligning with previous findings that EF performance is frequently associated with frontal lobe function [58, 59]. Component 9, exhibiting both univariate and multivariate association to EF, further supported this by showing high loadings on GM regions in frontotemporal regions, as well on the connecting WM tracts between these regions and the middle and superior frontal gyrus.

To the best of our knowledge, this study marks the first instance where joint-ICA has been performed in an MS cohort. We show that for some cognitive outcomes, joint-ICA components are more closely associated with cognitive measures than single-tissue ICA components, highlighting the potential value of this multimodal fusion approach in studying MS in relation to cognition. However, it should be kept in mind that the overall adjusted R^2^ were not consistently higher for models with joint-ICA components. Hence, the present results tell us about patterns of tissue features being relevant to outcomes, not that features involving GM and WM are generally more clinically significant. Furthermore, even using such complicated methods, only about a third of clinical variance was accounted for by either single-tissue or joint-ICA measures. It was beyond the scope of the present study to try to optimize the ICA to explain variability in clinical outcomes, or assess their independent contributions to clinical outcomes alongside conventional MRI measures (such as whole-brain atrophy or WM lesion loads). Future research could investigate whether the inclusion of additional MRI measures of tissue-specific pathology significantly improves outcomes, while further exploring the added value of a joint-ICA approach in explaining both pathological mechanisms and their assessment in clinical studies. The diversity of the cohort of people with MS spanning all clinical phenotypes, with a wide spread of clinical outcomes, was a key strength. However, a limitation was the lack of healthy controls, which prevented us from determining if there was CI, or if associations between ICA components and clinical outcomes were disease-related rather than normal variations between individuals. Future studies including healthy controls are needed to resolve these questions. In addition, the size of the progressive MS subgroups was relatively small (which, as would be expected from the natural history, also differed in terms of age, disease duration, EDSS and cognitive measures). Given this, the between-group comparisons, although clinically plausible, should be interpreted with caution. A larger and even more diverse cohort, including more people with progressive MS and controls, may be able to identify additional clinically relevant shared patterns of pathology. Furthermore, there were insufficient people in the cohort for cross-validation to test component stability, however, single-tissue GM ICA findings did replicate previous work, suggesting that the cohort was sufficiently large to yield reliable results. It is also reassuring to note that from the joint-ICA components, we found eight GM patterns that overlapped with stable GM components reported by earlier work [14] in a substantially larger cohort.

In conclusion, GM atrophy and WM structural integrity are both relevant to cognitive outcomes in MS, albeit to differing degrees dependent on the specific function being assessed. For visual memory and EF, it was the combination of GM and WM features that best explained outcomes, while for working memory, WM patterns had a dominant effect. This suggests that cognitive functions are not equally vulnerable to the same tissue features and that when seeking to explain clinical outcomes in MS, or other diseases that affect both GM and WM, single-tissue ICA and joint-ICA should both be used, as either in isolation would offer a significant incomplete assessment. The present analysis is based on cross-sectional data, however, in future work it would be interesting to assess longitudinal changes using a joint-ICA approach, to determine if GM and WM elements change sequentially or in parallel, and whether one element might predict subsequent functional changes while another mirrors it. A further follow-up of the present cohort has just been completed and will provide an opportunity to explore longitudinal changes, albeit with the methodological challenges of running longitudinal ICA rather than sequential cross-sectional analyses (for example accounting for changes in MRI scanner hardware and sequences). This can potentially offer valuable insights into the sequence of pathological events in networks and their translations into clinical outcomes.

## Supplementary Information

Below is the link to the electronic supplementary material.

Supplementary file1 (DOCX 4526 KB)

## Data Availability

The data that support the findings of this study can be requested from the corresponding author.
