# Supplementary material for: Explaining cognitive function in multiple sclerosis through networks of grey and white matter features: a joint independent component analysis
Source: J Neurol. 2025 Jan 15;272(2):142. doi: 10.1007/s00415-024-12795-2 (PMC11735591; doi:10.1007/s00415-024-12795-2)
Supplement: Supplementary file 1 — Supplementary file1 (DOCX 4526 KB) [file 415_2024_12795_MOESM1_ESM.docx]

**Title: Explaining cognitive function in multiple sclerosis through networks of grey and white matter features: A joint independent component analysis**

**Journal: Journal of Neurology**

**Authors:** Senne B. Lageman^1,2^, Amy Jolly^1^, Nitin Sahi^1^, Ferran Prados^1,3,4^, Baris Kanber^1,3^, Arman Eshaghi^1^, Carmen Tur^1,5^, Cyrus Eierud^6^, Vince D. Calhoun^6^, Menno M. Schoonheim^7^, Declan T. Chard^1,8^

**Affiliations:**

1. NMR Research Unit, Queen Square MS Centre, Department of Neuroinflammation, UCL Queen Square Institute of Neurology, Faculty of Brain Sciences, University College London, UK
2. Alzheimer Center Amsterdam, Neurology, Vrije Universiteit Amsterdam, Amsterdam UMC location VUmc, Amsterdam, The Netherlands
3. Department of Medical Physics and Biomedical Engineering, Centre for Medical Image Computing, UCL, London, UK.
4. e-Health Centre, Universitat Oberta de Catalunya, Barcelona, Spain.
5. Multiple Sclerosis Centre of Catalonia (CEMCAT), Vall d'Hebron Barcelona Hospital Campus, Barcelona, Spain.
6. Tri-Institutional Center for Translational Research in Neuroimaging and Data Science (TReNDS), Georgia Institute of Technology, Georgia State University, Emory University, Atlanta, GA, United States.
7. MS Center Amsterdam, Anatomy and Neurosciences, Vrije Universiteit Amsterdam, Amsterdam Neuroscience, Amsterdam University Medical Centers, Location VUmc, Amsterdam, The Netherlands
8. National Institute for Health Research (NIHR) University College London Hospitals (UCLH) Biomedical Research Centre, UK

**Correspondence:** Declan T. Chard, Russell Square House, 10-12 Russell Square, Queen Square MS Centre, Department of Neuroinflammation, UCL Queen Square Institute of Neurology, Faculty of Brain Sciences, University College London, London, UK WC1B 5EH, [d.chard@ucl.ac.uk](mailto:d.chard@ucl.ac.uk)

**Supplementary materials**

**Table S1. List of GM ROIs from the Desikan-Killiany-Tourville atlas**

| Nucleus Accumbens (L,R) |  | Middle Frontal Gyrus (L,R) |
| --- | --- | --- |
| Amygdala (L,R) |  | Middle Occipital Gyrus (L,R) |
| Caudate Nucleus (L,R) |  | Medial Orbital Gyrus (L,R) |
| CerebellumExterior (L,R) |  | Medial segment of the Postcentral Gyrus (L,R) |
| Hippocampus (L,R) |  | Medial segment of the Precentral Gyrus (L,R) |
| Pallidum (L,R) |  | Medial segment of the Superior Frontal Gyrus (L,R) |
| Putamen (L,R) |  | Middle Temporal Gyrus (L,R) |
| Thalamus (L,R) |  | Occipital Pole (L,R) |
| Ventral Diencephalon (L,R) |  | Occipital Fusiform Gyrus (L,R) |
| CerebellarVermalLobulesI-V |  | Opercular part of the Inferior Frontal Gyrus (L,R) |
| CerebellarVermalLobulesVI-VII |  | Orbital part of the Inferior Frontal Gyrus (L,R) |
| CerebellarVermalLobulesVIII-X |  | Posterior Cingulate Gyrus (L,R) |
| Basal Forebrain (L,R) |  | Precuneus (L,R) |
| Anterior Cingulate Gyrus (L,R) |  | Parahippocampal Gyrus (L,R) |
| Anterior Insula (L,R) |  | Posterior Insula (L,R) |
| Anterior Orbital Gyrus (L,R) |  | Parietal Operculum (L,R) |
| Angular Gyrus (L,R) |  | Postcentral Gyrus (L,R) |
| Calcarine Cortex (L,R) |  | Posterior Orbital Gyrus (L,R) |
| Central Operculum (L,R) |  | Planum Polare (L,R) |
| Cuneus (L,R) |  | Precentral Gyrus (L,R) |
| Entorhinal Area (L,R) |  | Planum Temporale (L,R) |
| Frontal Operculum (L,R) |  | Subcallosal Area (L,R) |
| Frontal Pole (L,R) |  | Superior Frontal Gyrus (L,R) |
| Fusiform Gyrus (L,R) |  | Supplementary Motor Cortex (L,R) |
| Gyrus Rectus (L,R) |  | Supramarginal Gyrus (L,R) |
| Inferior Occipital Gyrus (L,R) |  | Superior Occipital Gyrus (L,R) |
| Inferior Temporal Gyrus (L,R) |  | Superior Parietal Gyrus (L,R) |
| Lingual Gyrus (L,R) |  | Superior Temporal Gyrus (L,R) |
| Lateral Orbital Gyrus (L,R) |  | Temporal Pole (L,R) |
| Middle Cingulate Gyrus (L,R) |  | Triangular part of the Inferior Frontal Gyrus (L,R) |
| Medial Frontal Cortex (L,R) |  | Transverse Temporal Gyrus (L,R) |

*Abbreviations*: GM – grey matter, L – left, R – right, ROI – region of interest


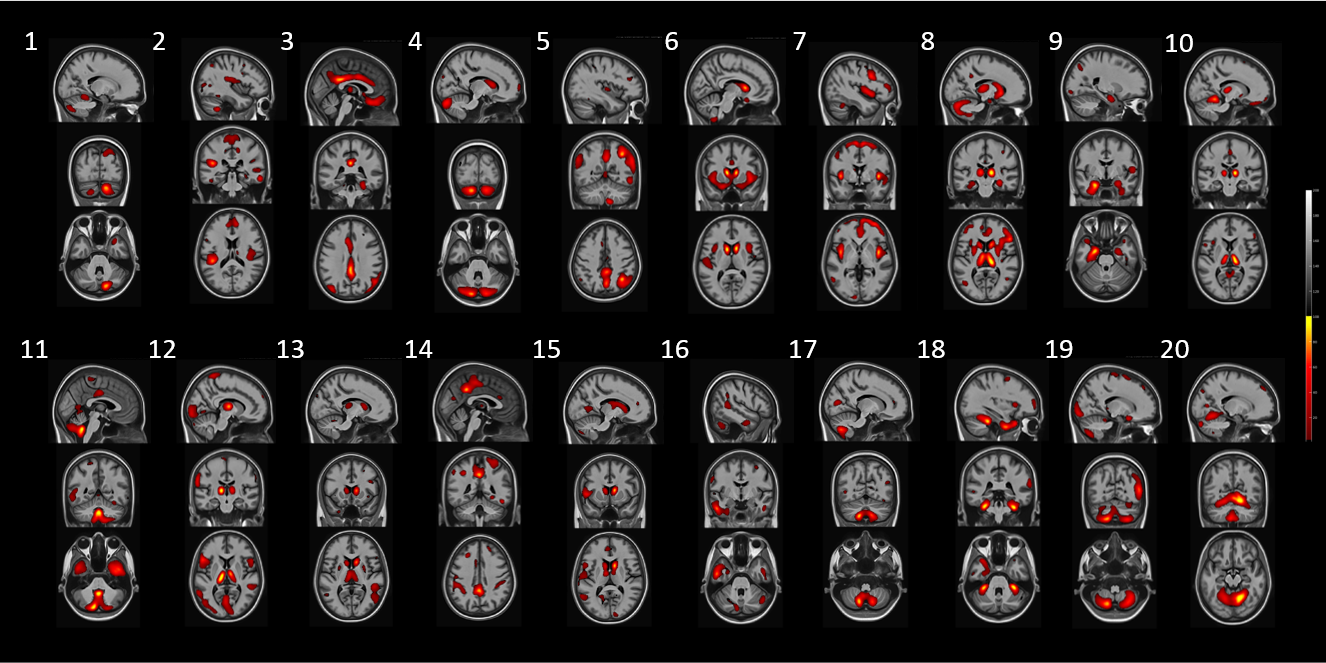


**Figure S1. The grey matter elements of joint-ICA components.** Components that either individually, or in the multivariate model, were associated with cognitive domains. Component 1 is a cerebellar-temporal component (cerebellar lobules I-V, caudate nucleus, central operculum and temporal gyri). Component 2 is a parietal-cerebellar component, involving mainly cerebellar lobules VIII-X, posterior cingulate, precuneus and parietal operculum. A cingulate-frontal pattern is displayed by component 3, encompassing mainly the posterior and middle cingulate, medial frontal cortex and gyrus rectus. Cerebellar-frontal component 4 involves the cerebellar exterior, frontal operculum and anterior insula. Component 5 is a posterior parietal-occipital pattern, spanning mainly the including posterior cingulate, angular gyrus, precuneus, lingual gyrus and amygdala. Component 6 resembles the default-mode network (precuneus, posterior cingulate and orbital gyrus) and salience network (anterior insula and anterior cingulate). Component 7 showed a right occipital-insular pattern (right occipital pole, calcarine cortex, superior occipital gyrus, bilateral anterior insula). A deep grey matter pattern is shown by component 8, encompassing mainly the thalamus, amygdala, putamen and caudate nucleus. Component 9 is a left temporal-cerebellar pattern, representing the cerebellar exterior and the left entorhinal area, hippocampus and parahippocampal gyrus. Component 10 is a visual-like network, involving the calcarine cortex, occipital fusiform gyrus and thalamus. Cerebellar-temporal component 11 displayed mainly the cerebellar lobules VIII-X, cerebellar exterior, temporal pole and inferior temporal gyrus. Component 12 is a thalamic-opercular pattern (thalamus, frontal operculum, opercular part of the inferior frontal gyrus). Component 13 encompassed mainly cingulate and DGM regions (anterior cingulate, caudate nucleus and thalamus). A cerebellar-inferior occipital pattern was shown by component 14, encompassing the cerebellum, occipital fusiform gyrus and inferior occipital gyrus. Component 15 is a cerebellar-occipital pattern (cerebellar lobules I-V, caudate nucleus, fusiform gyrus, posterior cingulate. A DGM-cortical pattern was shown by component 16 (thalamus, putamen, accumbens, precuneus, posterior insula). Component 17 is a cerebellar-temporal-cingulate pattern, involving mainly the cerebellum, transverse temporal gyrus and posterior cingulate. Component 18 is an opercular-occipital component (frontal operculum, opercular part of the inferior frontal gyrus, cuneus and calcarine cortex). Occipital-frontal component 19 involves mainly the occipital pole, calcarine cortex and subcallosal area. Component 20 is a cerebellar-temporal-occipital pattern, encompassing the cerebellum, lingual gyrus and middle temporal gyrus. *Abbreviations*: ICA – independent component analysis

**
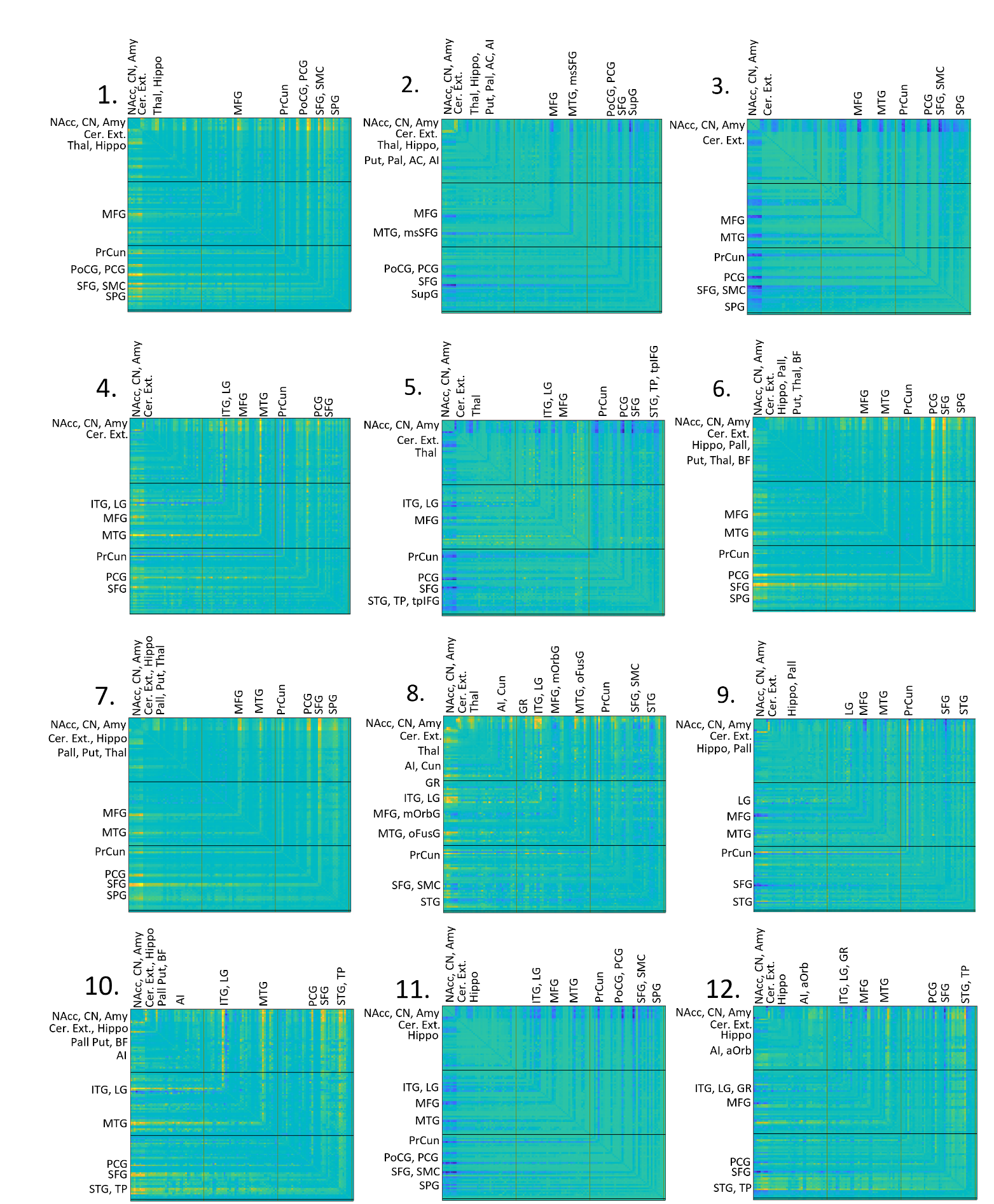
**

**
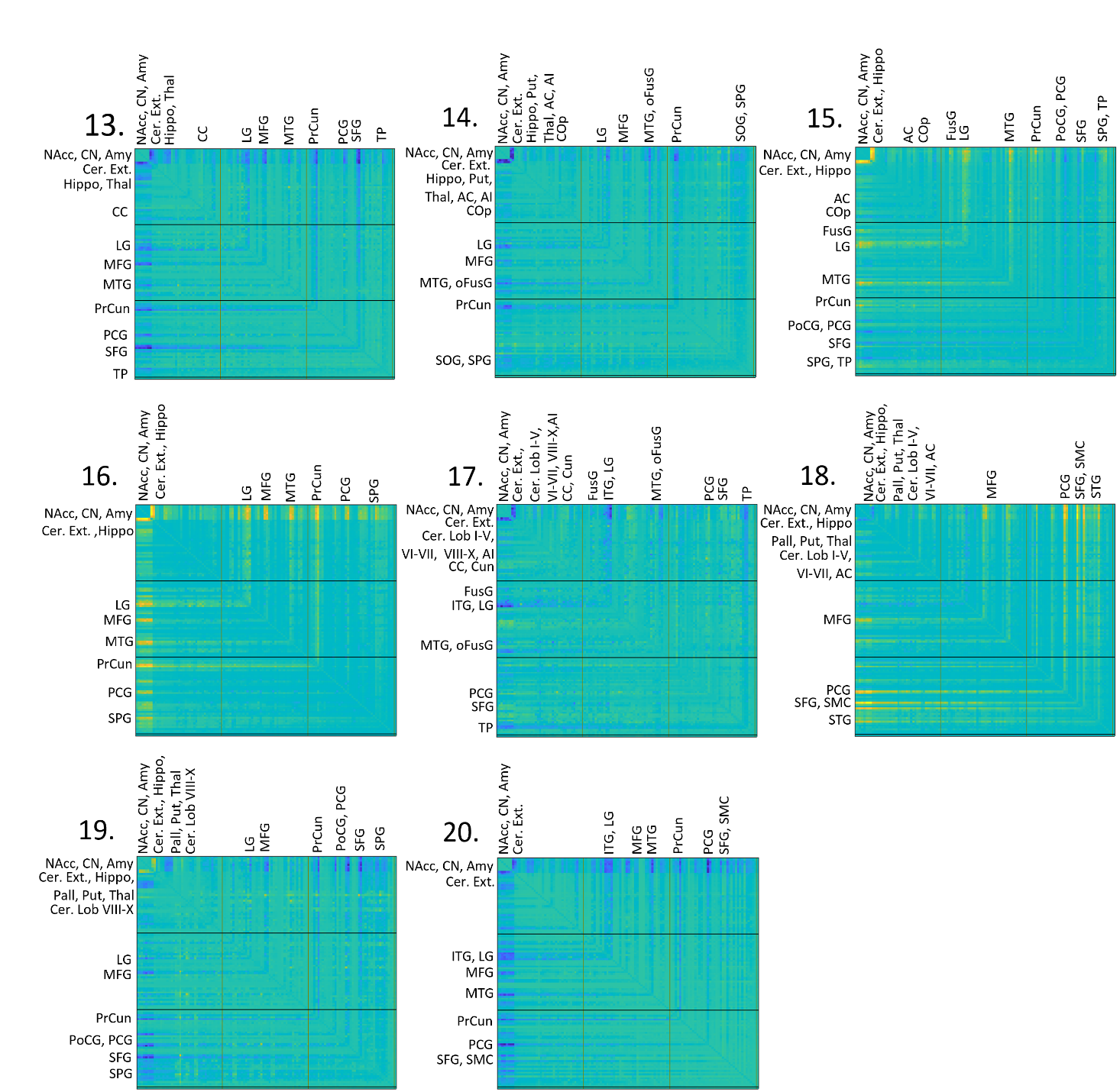
**

**Figure S2. The white matter elements of the joint-ICA components.** WM connectomes are shown with their node-to-node connections within the 99% confidence interval labeled. Component 1 shows WM connections between the amygdala, nucleus accumbens (NAcc), caudate nucleus (CN) and cerebellar exterior (CerExt) to the thalamus, frontal and motor areas. The WM connections of component 2 are predominantly between the limbic and DGM regions to frontal and parietal structures. For component 3 WM connections between amygdala, NAcc, CN and CerExt and precuneus and frontal regions showed highest loadings. Component 4 displayed a WM patterns from amygdala, NAcc, CN, CerExt, to primarily temporal regions, the precuneus and lingual gyrus (LG). The highest loadings of component 5 were on connections between amygdala, NAcc, CN and CerExt to frontal regions, thalamus and precuneus. Component 6 shows the highest loading WM connections between connecting several DGM and cerebellar structures with specifically the precentral and superior frontal gyrus. Component 7 has the highest loadings on connections between the amygdala, NAcc, CN and CerExt to frontal and parietal regions. WM connections of component 8 with high loadings came from the amygdala, striatum and cerebellum to temporal regions, parietal and occipital regions. Component 9 shows the highest loading connections between the amygdala, NAcc, CN and CerExt, precuneus, frontal and temporal regions. A WM pattern between the DGM structures, cerebellum, temporal regions, SFG and lingual gyrus was shown by component 10. Component 11 displays a WM pattern of connections between the amygdala, NAcc, CN and CerExt to frontal, parietal and temporal regions. Component 12 shows the highest loading connections between the amygdala, NAcc, CN and CerExt to frontal and temporal structures and the lingual gyrus. The WM connections of component 13 were loaded on most between DGM structures, cerebellum, precuneus, frontal and occipital regions. Component 14 showed high loading connections between amygdala, NAcc, CN and cerebellum, lingual gyrus, parietal and temporal regions. Component 15 involved connections between the caudate, cerebellum, posterior parietal and occipital regions. WM connections in component 16 were between the amygdala, striatum and cerebellum, precuneus, lingual gyrus, temporal and frontal regions. Component 17 showed a pattern of connections present between the amygdala, NAcc, CN and cerebellum, temporal and occipital structures. Component 18 involved mainly connections between several DGM structures, the cerebellum and frontal regions and the supplementary motor cortex. The WM pattern of component 19 shows connections between DGM, cerebellum, frontal and parietal regions. Component 20 involves the highest connections between the amygdala, NAcc, CN and CerExt, precuneus, lingual gyrus and temporal regions. *Abbreveations*: AC – anterior cingulate, AI – anterior insula, Amy – Amygdala, aOrbG – anterior orbital gyrus, BF – basal forebrain, CC – calcarine cortex, CerExt – cerebellum exterior, CerLob – cerebellar lobules, CN – caudate nucleus, COp – central operculum, Cun – cuneus, FOp – frontal operculum, FusG – fusiform gyrus, Hippo – hippocampus, GR – gyrus rectus, ICA – independent component analysis, IOG – inferior occipital gyrus, ITG – inferior temporal gyrus, LG – lingual gyrus, MC – middle cingulate, MFG – middle frontal gyrus, mOrbG – medial orbital gyrus, msSFG – medial segment of the superior frontal gyrus, MTG – middle temporal gyrus, NAcc – nucleus accumbens, OFusG – occipital fusiform gyrus, OP – occipital pole, opIFG – opercular part of the inferior frontal gyrus, OrbG – orbital gyrus, Pall - pallidum, PCG – precentral gyrus, PoCG – postcentral gyrus, POp – parietal operculum, PrCun - precuneus, Put - putamen, SCA – subcallocal area, SFG – superior frontal gyrus, SMC – supplementary motor cortex, SOG – superior occipital gyrus, SPG – superior parietal gyrus, STG – superior temporal gyrus, SupG – supramarginal gyrus, Thal - thalamus, TP – temporal pole, tpIFG – triangular part of the inferior frontal gyrus, TTG – transverse temporal gyrus.

**Table S2. List of all joint-ICA components with their corresponding GM ROIs and top 0.1% WM connections.**

|  | **GM ROIs** | **WM connections** | | |
| --- | --- | --- | --- | --- |
| Comp1 | Caudate nucleus↓, cerebellar lobules I-V↓, cerebellum exterior↓, superior parietal lobe↓, posterior insula↓, planum polare↓, central and frontal operculum↓, inferior↓, middle↓, transverse↓ and superior temporal gyrus↓, putamen↓, pallidum↓, lingual gyrus↓ and calcarine cortex↓ | Left nucleus accumbens | - | Right precentral gyrus↓ |
|  |  | Left amygdala | - | Right precentral gyrus↓ |
|  |  | Left caudate nucleus | - | Right precentral gyrus↓ |
|  |  | Left caudate nucleus | - | Right superior frontal gyrus↓ |
|  |  | Right cerebellar exterior | - | Right precentral gyrus↓ |
|  |  | Right cerebellar exterior | - | Right superior frontal gyrus↓ |
|  |  | Left cerebellar exterior | - | Right precentral gyrus↓ |
|  |  | Left cerebellar exterior | - | Right superior frontal gyrus↓ |
| Comp2 | Precuneus↑, posterior cingulate↑, cerebellar exterior↑, cerebellar lobules VIII-X↑, transverse temporal, postcentral gyrus medial segment↓, medial frontal cortex↑, parietal operculum↓, planum temporale↑, and anterior cingulate↑ | Left nucleus accumbens | - | Right superior frontal gyrus↑ |
|  |  | Right amygdala | - | Right superior frontal gyrus↑ |
|  |  | Left amygdala | - | Right superior frontal gyrus↑ |
|  |  | Right caudate nucleus | - | Right superior frontal gyrus↑ |
|  |  | Left caudate nucleus | - | Right superior frontal gyrus↑ |
|  |  | Right cerebellar exterior | - | Right superior frontal gyrus↑ |
|  |  | Left cerebellar exterior | - | Left middle frontal gyrus↑ |
|  |  | Left cerebellar exterior | - | Right superior frontal gyrus↑ |
| Comp3 | Frontal medial cortex↓, posterior↑ and middle cingulate↑, putamen↓, pallidum↑, thalamus↑, middle temporal gyrus↓, occipital fusiform gyrus↓, gyrus rectus↑, fusiform gyrus↑, middle occipital gyrus↓, lingual gyrus↓ and inferior temporal gyrus↑ | Left amygdala | - | Right precentral gyrus↓ |
|  |  | Left amygdala | - | Right superior frontal gyrus↓ |
|  |  | Left caudate | - | Right precentral gyrus↓ |
|  |  | Left caudate | - | Right superior frontal gyrus↓ |
|  |  | Right cerebellar exterior | - | Right precentral gyrus↓ |
|  |  | Right cerebellar exterior | - | Right superior frontal gyrus↓ |
|  |  | Left cerebellar exterior | - | Right precentral gyrus↓ |
|  |  | Left cerebellar exterior | - | Right superior frontal gyrus↓ |
| Comp4 | Left frontal↑ and central operculum↑, left anterior insula↑, thalamus↓, putamen↑, right caudate nucleus↓, cerebellar exterior↑, inferior frontal gyrus (pars triangular↑, opercular↑, orbital↑), inferior temporal gyrus↑, posterior cingulate↑ and left occipital fusiform gyrus↑ | Right amygdala | - | Right middle temporal gyrus↑ |
|  |  | Left amygdala | - | Right middle temporal gyrus↑ |
|  |  | Left amygdala | - | Right superior frontal gyrus↑ |
|  |  | Right caudate | - | Right middle temporal gyrus↑ |
|  |  | Left caudate | - | Right middle temporal gyrus↑ |
|  |  | Right cerebellum exterior | - | Right middle temporal gyrus↑ |
|  |  | Left cerebellum exterior | - | Right middle temporal gyrus↑ |
|  |  | Right cerebellum exterior | - | Right middle temporal gyrus↑ |
| Comp5 | Posterior cingulate↓, angular gyrus↑, precuneus↓, hippocampus↓, amygdala↓, parietal superior gyrus↓, lingual gyrus↓, fusiform gyrus↑, cerebellar exterior↓, anterior cingulate↓, medial orbital gyrus↓, superior temporal gyrus↓, parahippocampal gyrus↓, subcallosal area↓, calcarine cortex↓, middle↓ and superior occipital gyrus↓, posterior orbital gyrus ↓ | Right nucleus accumbens | - | Left precuneus↑ |
|  |  | Right nucleus accumbens | - | Left precentral gyrus↑ |
|  |  | Right nucleus accumbens | - | Right superior frontal gyrus↑ |
|  |  | Left caudate nucleus | - | Left precentral gyrus↑ |
|  |  | Right cerebellar exterior | - | Left precentral gyrus↑ |
|  |  | Right cerebellar exterior | - | Right superior frontal gyrus↓ |
|  |  | Left cerebellar exterior | - | Left precentral gyrus↑ |
|  |  | Left cerebellar exterior | - | Right superior frontal gyrus↓ |
| Comp6 | Caudate nucleus↓, precuneus↓, posterior cingulate↓, anterior insula↓, anterior cingulate↓, posterior↓, medial↓ and lateral↓ orbital gyrus, frontal operculum↓, inferior frontal orbital gyrus↑, hippocampus↓ and middle occipital gyrus↓ | Right nucleus accumbens | - | Right precentral gyrus↑ |
|  |  | Left nucleus accumbens | - | Right precentral gyrus↑ |
|  |  | Right amygdala | - | Right precentral gyrus↑ |
|  |  | Right amygdala | - | Right superior frontal gyrus↑ |
|  |  | Left amygdala | - | Right precentral gyrus↑ |
|  |  | Left amygdala | - | Right superior frontal gyrus↑ |
|  |  | Right cerebellar exterior | - | Right precentral gyrus↑ |
|  |  | Left cerebellar exterior | - | Right precentral gyrus↑ |
| Comp7 | Right occipital pole↓, right superior occipital gyrus↓, right calcarine cortex↓, anterior↓ and posterior insula↓, cuneus↓, precuneus↓, right anterior orbital gyrus↓, frontal pole↓, opercular part inferior frontal gyrus↓, middle frontal gyrus↓ and lingual gyrus↓ | Right nucleus accumbens | - | Left superior frontal gyrus↓ |
|  |  | Right caudate nucleus | - | Right superior frontal gyrus↓ |
|  |  | Right caudate nucleus | - | Left superior frontal gyrus↓ |
|  |  | Left caudate nucleus | - | Right superior frontal gyrus↓ |
|  |  | Right cerebellar exterior | - | Right superior frontal gyrus↓ |
|  |  | Right cerebellar exterior | - | Left superior frontal gyrus↓ |
|  |  | Left cerebellar exterior | - | Right superior frontal gyrus↓ |
|  |  | Left cerebellar exterior | - | Left superior frontal gyrus↓ |
| Comp8 | Thalamus↓, caudate nucleus↓, putamen↓, amygdala↓, nucleus accumbens↓, hippocampus↓, anterior insula↓, cerebellum lobules VIII-X↓, frontal operculum↓, triangular part inferior frontal gyrus↓, anterior↓ and posterior cingulate↓ and pallidum↓ | Right amygdala | - | Left cerebellar exterior↓ |
|  |  | Right amygdala | - | Left cuneus↓ |
|  |  | Right amygdala | - | Right inferior temporal gyrus↓ |
|  |  | Right amygdala | - | Left lingual gyrus↓ |
|  |  | Right amygdala | - | Left precuneus↓ |
|  |  | Left amygdala | - | Left precuneus↓ |
|  |  | Right caudate nucleus | - | Left lingual gyrus↓ |
|  |  | Right anterior insula | - | Left lingual gyrus↓ |
| Comp9 | Left entorhinal area↓, left parahippocampal gyrus↓, amygdala↓, hippocampus↓, middle temporal gyrus↓, precentral gyrus medial segment↓, cerebellum exterior↓, caudate nucleus↓, temporal pole↓ and fusiform gyrus↓ | Right nucleus accumbens | - | Left middle frontal gyrus↓ |
|  |  | Right nucleus accumbens | - | Left precuneus↓ |
|  |  | Right nucleus accumbens | - | Left precentral gyrus↓ |
|  |  | Right nucleus accumbens | - | Left superior frontal gyrus↓ |
|  |  | Left nucleus accumbens | - | Left superior frontal gyrus↓ |
|  |  | Right amygdala | - | Left middle frontal gyrus↓ |
|  |  | Right amygdala | - | Left superior frontal gyrus↓ |
|  |  | Left amygdala | - | Left superior frontal gyrus↓ |
| Comp10 | Calcarine cortex↓, occipital fusiform gyrus↓, thalamus↓, lingual gyrus↓, cuneus↓, precuneus↓, inferior occipital gyrus↓, cerebellar lobules I-V↓, planum temporale↓, transverse↓ and superior temporal gyrus↓, gyrus rectus↓ and posterior cingulate↓ | Right nucleus accumbens | - | Right inferior temporal gyrus↑ |
|  |  | Right amygdala | - | Right inferior temporal gyrus↑ |
|  |  | Right amygdala | - | Right middle temporal gyrus↑ |
|  |  | Left amygdala | - | Right inferior temporal gyrus↑ |
|  |  | Left amygdala | - | Right lingual gyrus↓ |
|  |  | Left caudate nucleus | - | Right inferior temporal gyrus↑ |
|  |  | Right cerebellar exterior | - | Right inferior temporal gyrus↑ |
|  |  | Left cerebellar exterior | - | Right inferior temporal gyrus↑ |
| Comp11 | Cerebellum VIII-X↓, cerebellum exterior↓, temporal pole↓, inferior↓, middle↓ and superior temporal gyrus↓, middle cingulate↓ and fusiform gyrus↓ | Right amygdala | - | Left precuneus↑ |
|  |  | Right amygdala | - | Right superior frontal gyrus↑ |
|  |  | Left amygdala | - | Right superior frontal gyrus↑ |
|  |  | Left caudate nucleus | - | Right superior frontal gyrus↑ |
|  |  | Right cerebellar exterior | - | Right precentral gyrus↑ |
|  |  | Right cerebellar exterior | - | Right superior frontal gyrus↑ |
|  |  | Left cerebellar exterior | - | Right precentral gyrus↑ |
|  |  | Left cerebellar exterior | - | Right superior frontal gyrus↑ |
| Comp12 | Thalamus↑, opercular part inferior frontal gyrus↑, medial segment postcentral gyrus↑, frontal operculum↑, lingual gyrus↑, hippocampus↑, posterior cingulate↑, superior↑ and middle temporal gyrus↑, amygdala↑, precuneus↑ and postcentral gyrus↑ | Right nucleus accumbens | - | Right cerebellar exterior↓ |
|  |  | Right nucleus accumbens | - | Left lingual gyrus↓ |
|  |  | Left nucleus accumbens | - | Right superior frontal gyrus↓ |
|  |  | Right amygdala | - | Left middle frontal gyrus↓ |
|  |  | Right amygdala | - | Right superior frontal gyrus↓ |
|  |  | Left amygdala | - | Right superior frontal gyrus↓ |
|  |  | Right cerebellar exterior | - | Right superior frontal gyrus↓ |
|  |  | Left cerebellar exterior | - | Right superior frontal gyrus↓ |
| Comp13 | Anterior cingulate↓, posterior cingulate↓, precuneus↓, caudate nucleus↑, cerebellar exterior↓, thalamus↓, lingual gyrus↓, medial segment superior frontal gyrus↓, calcarine cortex↓, cerebellum exterior↓ and medial segment precentral gyrus↓ | Right nucleus accumbens | - | Left superior frontal gyrus↑ |
|  |  | Right amygdala | - | Right cerebellar exterior↑ |
|  |  | Right amygdala | - | Left superior frontal gyrus↑ |
|  |  | Left amygdala | - | Right cerebellar exterior↑ |
|  |  | Right caudate nucleus | - | Left superior frontal gyrus↑ |
|  |  | Right cerebellar exterior | - | Left superior frontal gyrus↑ |
|  |  | Left cerebellar exterior | - | Left precuneus↑ |
|  |  | Left cerebellar exterior | - | Left superior frontal gyrus↑ |
| Comp14 | Occipital fusiform gyrus↓, cerebellar vermal lobules I-V↓and VIII-X↓, posterior cingulate↓, inferior occipital gyrus↑, medial segment precentral gyrus↓, lingual gyrus↑ and cerebellar exterior↓ | Left nucleus accumbens | - | Left cerebellar exterior↓ |
|  |  | Right amygdala | - | Right cerebellar exterior↓ |
|  |  | Left amygdala | - | Right cerebellar exterior↓ |
|  |  | Right caudate nucleus | - | Right cerebellar exterior↓ |
|  |  | Right caudate nucleus | - | Left cerebellar exterior↓ |
|  |  | Left caudate nucleus | - | Right cerebellar exterior↓ |
|  |  | Left caudate nucleus | - | Left cerebellar exterior↓ |
|  |  | Right cerebellar exterior | - | Left cerebellar exterior↓ |
| Comp15 | Cerebellar lobules I-V↓, caudate nucleus↓, fusiform gyrus↓, frontal↓ and central operculum↓, posterior cingulate↓, cerebellar exterior↓, precentral gyrus↓ and lingual gyrus↓ | Right nucleus accumbens | - | Right cerebellar exterior↑ |
|  |  | Left nucleus accumbens | - | Right cerebellar exterior↑ |
|  |  | Left nucleus accumbens | - | Left cerebellar exterior↑ |
|  |  | Right amygdala | - | Right cerebellar exterior↑ |
|  |  | Right amygdala | - | Left cerebellar exterior↑ |
|  |  | Left amygdala | - | Right cerebellar exterior↑ |
|  |  | Right caudate nucleus | - | Right cerebellar exterior↑ |
|  |  | Left caudate nucleus | - | Right cerebellar exterior↑ |
| Comp16 | Thalamus↓, precuneus↓, posterior insula↓, putamen↓, lingual gyrus↓, nucleus accumbens↓, subcallosal area↓, cerebellar lobules I-V↓, calcarine cortex↓, cuneus↓, medial orbital gyrus↓, parietal operculum↓, posterior cingulate↓, inferior↓ and middle temporal gyrus↓ and pallidum↓ | Left nucleus accumbens | - | Right cerebellar exterior↑ |
|  |  | Right amygdala | - | Right cerebellar exterior↑ |
|  |  | Right amygdala | - | Left precuneus↑ |
|  |  | Left amygdala | - | Right cerebellar exterior↑ |
|  |  | Right caudate nucleus | - | Right cerebellar exterior↑ |
|  |  | Right caudate nucleus | - | Left precuneus↑ |
|  |  | Left caudate nucleus | - | Right cerebellar exterior↑ |
|  |  | Right cerebellar exterior | - | Left precuneus↑ |
| Comp17 | Cerebellar lobules I – V ↓, VIII-X↓, VI – VII↓, cerebellar exterior↓, posterior cingulate↓, temporal transverse gyrus↓, postcentral gyrus↓, middle cingulate↓, orbital part inferior frontal gyrus↓, central operculum↓, supramarginal gyrus↓ and planum temporale↓ | Right nucleus accumbens | - | Left lingual gyrus↓ |
|  |  | Right amygdala | - | Right cerebellar exterior↓ |
|  |  | Right amygdala | - | Left lingual gyrus↓ |
|  |  | Right caudate nucleus | - | Right cerebellar exterior↓ |
|  |  | Left caudate nucleus | - | Right cerebellar exterior↓ |
|  |  | Left caudate nucleus | - | Left lingual gyrus↓ |
|  |  | Right cerebellar exterior | - | Left cerebellar exterior↓ |
|  |  | Right cerebellar exterior | - | Left lingual gyrus↓ |
| Comp18 | Cuneus↓, frontal operculum↓, opercular part inferior frontal gyrus↓, gyrus rectus↓, precuneus↓, fusiform gyrus↓, left entorhinal area↓, temporal pole↓, superior occipital gyrus↓, medial frontal cortex↓ and calcarine cortex↓ | Left nucleus accumbens | - | Right superior frontal gyrus↓ |
|  |  | Right amygdala | - | Right superior frontal gyrus↓ |
|  |  | Left amygdala | - | Right superior frontal gyrus↓ |
|  |  | Left caudate nucleus | - | Right precentral gyrus↓ |
|  |  | Left caudate nucleus | - | Right superior frontal gyrus↓ |
|  |  | Right cerebellar exterior | - | Right precentral gyrus↓ |
|  |  | Right cerebellar exterior | - | Right superior frontal gyrus↓ |
|  |  | Left cerebellar exterior | - | Right superior frontal gyrus↓ |
| Comp19 | Calcarine cortex↓, occipital pole↓, inferior↓ and middle occipital gyrus↓, lingual gyrus↑, subcallosal area↑, caudate nucleus↑, inferior↑ and middle temporal gyrus↑, cerebellar exterior↑, medial orbital gyrus↑ and occipital fusiform gyrus↓ | Right nucleus accumbens | - | Left superior frontal gyrus↓ |
|  |  | Left amygdala | - | Left superior frontal gyrus↓ |
|  |  | Right caudate nucleus | - | Left precentral gyrus↓ |
|  |  | Right caudate nucleus | - | Left superior frontal gyrus↓ |
|  |  | Right cerebellar exterior | - | Left precentral gyrus↓ |
|  |  | Right cerebellar exterior | - | Left superior frontal gyrus↓ |
|  |  | Left cerebellar exterior | - | Left precentral gyrus↓ |
|  |  | Left cerebellar exterior | - | Left superior frontal gyrus↓ |
| Comp20 | Cerebellar lobules I-V↑, VI-VII↑, VIII-X↑, cerebellar exterior↑, lingual gyrus↑, inferior↑ and middle occipital↑, inferior↑ and middle temporal gyrus↑, opercular part inferior frontal gyrus↑, anterior cingulate↑ and fusiform gyrus↑ | Left nucleus accumbens | - | Right precentral gyrus↓ |
|  |  | Right amygdala | - | Left precuneus↓ |
|  |  | Right amygdala | - | Right precentral gyrus↓ |
|  |  | Left amygdala | - | Right middle temporal gyrus↓ |
|  |  | Left amygdala | - | Right precentral gyrus↓ |
|  |  | Left caudate nucleus | - | Right precentral gyrus↓ |
|  |  | Right cerebellar exterior | - | Right precentral gyrus↓ |
|  |  | Left cerebellar exterior | - | Right precentral gyrus↓ |

Parcellation masks were overlayed with the GM part of components, and the connectomes were labeled to identify the regions and connections in each component. Volumes of GM regions and number of streamlines and FA for WM connections were correlated with component loadings to identify the directionality of the tissue-values of regions (↑ positive correlation or ↓ negative correlation). GM regions are described bilaterally unless mentioned otherwise. *Abbreviations*: Comp – component, FA – fractional anisotropy, GM – grey matter, ICA – Independent component analysis, ROI – region of interest, WM – white matter.

**Table S3. Component comparison between groups**

|  | RRMS (n=53) | SPMS (n=22) | PPMS (n=14) |
| --- | --- | --- | --- |
| Component 4 | 0.15 | 0.24^^^ * | 0.12 |
| Component 6 | 0.20 | 0.33^^^ | 0.30 |
| Component 20 | 0.09 | 0.08 | 0.20^^^ #^ |

Data are presented as mean. Differences between groups were compared using ANCOVA with age and gender as covariates and Tukey HSD post-hoc when appropriate. ^^^p=0.05, ^^^^p<0.01 compared to RRMS, ^#^p<0.05 compared to SPMS, *p<0.05 compared to PPMS. *Abbreviations*: PPMS – Primary Progressive Multiple Sclerosis, RRMS – Relapse-Remitting Multiple Sclerosis, SPMS – Secondary Progressive Multiple Sclerosis

**Table S4. Association between joint-ICA component loadings and tissue values. All p-values are corrected for multiple comparison using FDR correction.**

|  | CGM | | DGM | | FA | | Streamlines | |
| --- | --- | --- | --- | --- | --- | --- | --- | --- |
|  | **r** | **p** | **r** | **p** | **r** | **p** | **r** | **p** |
| Comp1 | 0.054 | 0.769 | 0.074 | 0.612 | 0.030 | 0.987 | -0.070 | 0.689 |
| Comp2 | 0.291 | 0.014 | 0.109 | 0.475 | 0.101 | 0.688 | 0.167 | 0.394 |
| Comp3 | 0.061 | 0.764 | 0.353 | 0.005 | 0.233 | 0.248 | 0.256 | 0.294 |
| Comp4 | 0.204 | 0.117 | 0.267 | 0.045 | 0.080 | 0.833 | 0.075 | 0.689 |
| Comp5 | 0.018 | 0.913 | 0.088 | 0.552 | 0.019 | 0.987 | -0.177 | 0.391 |
| Comp6 | 0.487 | 0.000 | 0.210 | 0.107 | 0.120 | 0.654 | 0.050 | 0.757 |
| Comp7 | 0.356 | 0.002 | 0.252 | 0.049 | 0.012 | 0.987 | 0.211 | 0.294 |
| Comp8 | 0.735 | 0.000 | 0.679 | 0.000 | 0.154 | 0.428 | 0.023 | 0.831 |
| Comp9 | 0.447 | 0.000 | 0.245 | 0.051 | 0.221 | 0.248 | 0.092 | 0.655 |
| Comp10 | 0.201 | 0.117 | 0.092 | 0.552 | 0.003 | 0.987 | 0.041 | 0.782 |
| Comp11 | 0.566 | 0.000 | 0.445 | 0.000 | 0.036 | 0.987 | -0.092 | 0.655 |
| Comp12 | -0.042 | 0.820 | 0.015 | 0.933 | 0.285 | 0.137 | -0.121 | 0.643 |
| Comp13 | -0.081 | 0.690 | 0.146 | 0.289 | -0.062 | 0.944 | 0.058 | 0.737 |
| Comp14 | 0.018 | 0.913 | 0.065 | 0.640 | 0.002 | 0.987 | 0.121 | 0.643 |
| Comp15 | 0.140 | 0.347 | 0.039 | 0.798 | 0.178 | 0.350 | 0.201 | 0.294 |
| Comp16 | 0.072 | 0.719 | 0.321 | 0.011 | 0.011 | 0.987 | 0.204 | 0.294 |
| Comp17 | 0.602 | 0.000 | 0.253 | 0.049 | 0.182 | 0.350 | 0.073 | 0.689 |
| Comp18 | 0.697 | 0.000 | 0.157 | 0.259 | 0.109 | 0.688 | 0.103 | 0.655 |
| Comp19 | 0.121 | 0.431 | 0.171 | 0.220 | 0.044 | 0.987 | 0.025 | 0.831 |
| Comp20 | 0.004 | 0.970 | 0.009 | 0.934 | 0.173 | 0.350 | 0.093 | 0.655 |

*Abbreviations:* CGM – cortical grey matter, Comp – component, DGM – deep grey matter, ICA – independent component analysis, ROI – region of interest, r – rho, WM – white matter.

**Table S5. Partial correlation values between joint-ICA component loadings and cognitive domains with age and gender as covariates. All p-values are corrected for multiple comparison using FDR correction.**

|  | IPS | | Vermem | | Vismem | | EF | | Workmem | |
| --- | --- | --- | --- | --- | --- | --- | --- | --- | --- | --- |
|  | **r** | **p** | **r** | **p** | **r** | **p** | **r** | **p** | **r** | **p** |
| Comp1 | -0.057 | 0.667 | 0.090 | 0.684 | -0.004 | 0.995 | 0.080 | 0.706 | 0.095 | 0.756 |
| Comp2 | 0.090 | 0.477 | -0.074 | 0.684 | 0.161 | 0.444 | 0.047 | 0.728 | 0.044 | 0.781 |
| Comp3 | -0.165 | 0.216 | -0.071 | 0.684 | 0.020 | 0.978 | 0.043 | 0.728 | 0.110 | 0.685 |
| Comp4 | -0.151 | 0.246 | 0.076 | 0.684 | 0.001 | 0.995 | 0.062 | 0.728 | 0.042 | 0.781 |
| Comp5 | 0.226 | 0.128 | 0.154 | 0.624 | 0.314 | 0.057 | 0.089 | 0.706 | -0.023 | 0.834 |
| Comp6 | -0.261 | 0.128 | -0.328 | 0.036 | -0.256 | 0.159 | 0.225 | 0.235 | 0.221 | 0.259 |
| Comp7 | 0.185 | 0.170 | 0.091 | 0.684 | 0.128 | 0.586 | 0.038 | 0.728 | 0.041 | 0.781 |
| Comp8 | 0.198 | 0.160 | 0.124 | 0.624 | 0.091 | 0.625 | 0.042 | 0.728 | 0.148 | 0.572 |
| Comp9 | -0.186 | 0.170 | -0.218 | 0.417 | -0.051 | 0.834 | 0.312 | 0.062 | 0.221 | 0.259 |
| Comp10 | -0.020 | 0.879 | 0.034 | 0.840 | 0.114 | 0.625 | 0.187 | 0.295 | 0.067 | 0.781 |
| Comp11 | 0.237 | 0.128 | 0.010 | 0.925 | 0.072 | 0.721 | 0.225 | 0.235 | 0.049 | 0.781 |
| Comp12 | 0.203 | 0.160 | 0.038 | 0.840 | 0.162 | 0.444 | 0.122 | 0.706 | -0.075 | 0.781 |
| Comp13 | 0.016 | 0.879 | 0.125 | 0.624 | 0.090 | 0.625 | 0.051 | 0.728 | 0.147 | 0.572 |
| Comp14 | -0.163 | 0.216 | -0.186 | 0.556 | -0.162 | 0.444 | 0.183 | 0.295 | 0.249 | 0.259 |
| Comp15 | -0.121 | 0.371 | -0.078 | 0.684 | 0.100 | 0.625 | -0.068 | 0.728 | 0.115 | 0.685 |
| Comp16 | -0.225 | 0.128 | -0.011 | 0.925 | -0.016 | 0.978 | 0.107 | 0.706 | 0.128 | 0.674 |
| Comp17 | -0.259 | 0.128 | -0.145 | 0.624 | 0.172 | 0.444 | 0.104 | 0.706 | 0.160 | 0.572 |
| Comp18 | 0.100 | 0.442 | -0.060 | 0.722 | 0.097 | 0.625 | 0.089 | 0.706 | 0.035 | 0.783 |
| Comp19 | -0.221 | 0.128 | -0.128 | 0.624 | 0.132 | 0.586 | 0.085 | 0.706 | 0.041 | 0.781 |
| Comp20 | -0.111 | 0.402 | -0.098 | 0.684 | 0.046 | 0.834 | 0.195 | 0.295 | -0.057 | 0.781 |

*Abbreviations:* Comp – component, EF – executive function, FDR – False Discovery Rate, ICA – independent component analysis, IPS – information processing speed, r – rho, vermem – verbal memory, vismem – visual memory, workmem – working memory
